# Can Allostery Be a Key Strategy for Targeting PTP1B in Drug Discovery? A Lesson from Trodusquemine

**DOI:** 10.3390/ijms24119621

**Published:** 2023-06-01

**Authors:** Rosanna Maccari, Rosaria Ottanà

**Affiliations:** Department of Chemical, Biological, Pharmaceutical and Environmental Sciences, University of Messina, Viale F. Stagno d’Alcontres 31, 98166 Messina, Italy; rottana@unime.it

**Keywords:** protein tyrosine phosphatase 1B, allosteric inhibition, trodusquemine, MSI-1436, diabetes, obesity, cancer, neurodegenerative diseases, structure–activity relationships, drug discovery

## Abstract

Protein tyrosine phosphatase 1B (PTP1B) is an enzyme crucially implicated in aberrations of various signaling pathways that underlie the development of different human pathologies, such as obesity, diabetes, cancer, and neurodegenerative disorders. Its inhibition can prevent these pathogenetic events, thus providing a useful tool for the discovery of novel therapeutic agents. The search for allosteric PTP1B inhibitors can represent a successful strategy to identify drug-like candidates by offering the opportunity to overcome some issues related to catalytic site-directed inhibitors, which have so far hampered the development of drugs targeting this enzyme. In this context, trodusquemine (MSI-1436), a natural aminosterol that acts as a non-competitive PTP1B inhibitor, appears to be a milestone. Initially discovered as a broad-spectrum antimicrobial agent, trodusquemine exhibited a variety of unexpected properties, ranging from antidiabetic and anti-obesity activities to effects useful to counteract cancer and neurodegeneration, which prompted its evaluation in several preclinical and clinical studies. In this review article, we provide an overview of the main findings regarding the activities and therapeutic potential of trodusquemine and their correlation with PTP1B inhibition. We also included some aminosterol analogues and related structure–activity relationships that could be useful for further studies aimed at the discovery of new allosteric PTP1B inhibitors.

## 1. Introduction

Protein tyrosine phosphorylation represents a fundamental event that is crucially involved in the regulation of numerous cellular functions, such as growth, differentiation, cell–cell communication, response to hormones, and gene transcription. The phosphorylation levels of protein tyrosine residues are dynamically regulated by the concerted activities of enzymes belonging to the families of protein tyrosine kinases (PTKs) and phosphotyrosine protein phosphatases (PTPs). In the last three decades, PTKs have been widely explored as molecular targets of new drugs, and several inhibitors of different PTKs are available on the market as therapeutic agents for the treatment of various human tumors [1]. In later years, PTPs were also recognized to be implicated in cellular signaling aberrations underlying the pathogenesis of several human diseases, such as metabolic and immune disorders, cancer, and neurodegenerative diseases. Therefore, certain PTPs emerged as valuable molecular targets for the development of novel therapeutic agents [2,3,4,5,6].

The human genome encodes more than 100 PTPs, comprising soluble cytoplasmatic enzymes and transmembrane receptor-like proteins [7]. Among them, PTP1B, a class I cysteine-based cytoplasmatic PTP, is considered the prototypical member of this enzyme family. Its extensive study allowed a large body of evidence to be acquired regarding its functions and involvement in different cell signaling pathways, as well as its implication in pathogenic mechanisms underlying the development of different human diseases [8,9,10].

On the basis of the considerable knowledge acquired on the structure, functions, and pathogenetic implications of PTP1B, this enzyme is considered a promising target for drug design. Although the search for PTP1B-targeting drugs has been studded with numerous challenges, PTP1B continues to attract great interest, due to the notable therapeutic potential of its inhibitors.

In this article, we provide an up-to-date review of the main findings reported in the literature on trodusquemine, one of the most promising PTP1B inhibitors, which exhibited several noteworthy properties in preclinical studies and also entered clinical trials. We believe that the knowledge acquired on this allosteric inhibitor can strongly contribute to both shed light on the possible therapeutic applications of PTP1B inhibitors and validate allostery as a key strategy for targeting this enzyme in drug development. In the discussion, some natural and synthetic analogues of trodusquemine and their structure–activity relationships, as well as a brief account of the current state of knowledge about PTP1B physiopathological functions, are also included.

## 2. PTP1B Functions and Involvement in Pathogenic Mechanisms Underlying Human Diseases

The most widely studied features of PTP1B are related to its pivotal functions in the regulation of glucose metabolism and energy homeostasis. In particular, PTP1B acts as a major negative regulator of both insulin and leptin signaling pathways by dephosphorylating specific tyrosine residues of proteins involved in the cellular response to these hormones [11,12,13,14,15].

Insulin signal transduction, which is essential for the control of glucose homeostasis and metabolism, is triggered by the interaction of the hormone with the insulin receptor (IR), which is a tyrosine kinase. As a result of insulin binding to the extracellular receptor subunits *α*, IR autophosphorylates specific tyrosine residues of its intracellular subunits *β*. Then, the activated IR phosphorylates different intracellular substrates, such as IRS proteins, which in turn activate other downstream intracellular components, such as PI3K and Akt proteins [16]. Insulin signaling is negatively controlled by the coordinated non-redundant actions of several PTPs, such as PTP1B, LMW-PTP, and TC-PTP [5,6,13,14]. Among them, PTP1B was shown to play a crucial role through the dephosphorylation of specific phosphotyrosine (pTyr) residues of subunits *β* and IRS proteins [14,17]. These actions result in the attenuation of insulin signaling, particularly in the liver, brain, skeletal muscle, and adipose tissues, where the regulation of glucose homeostasis is a fundamental function [13,15,18,19,20].

Moreover, PTP1B acts as a negative regulator of leptin signal transduction by dephosphorylating Janus 2 kinase (JAK2), which is associated with the leptin receptor, and consequently preventing the activation of the downstream signal transducer and activator of transcription 3 (STAT3). Leptin, which is produced by adipocytes, acts in the hypothalamus by suppressing appetite and stimulating energy expenditure, thus exerting crucial control over body weight [11,12]. This adipokine also participates in the regulation of intestinal functions such as nutrient absorption and immune actions [21].

Although insulin and leptin are mediators of distinct cellular responses, both hormones play key roles in the brain through the activation of the intracellular PI3K/Akt and JAK2/STAT3 signal transduction pathways, respectively, thus cooperating as regulators of energy expenditure, glucose homeostasis, and neuronal excitability. In fact, both insulin and leptin receptors mediate anorexigenic effects by suppressing the activity of hypothalamic orexigenic neuropeptide Y (NPY)/agouti-related peptide (AgRP) neurons and stimulating anorexigenic pro-opiomelanocortin (POMC) neurons located in the arcuate nucleus. The insulin signal can also propagate to other regions of the hypothalamus and amygdala, from which it can exert a crucial control on glucose and energy homeostasis in peripheral tissues such as white and brown adipose tissues, the pancreas, muscle, and the liver [9,13,20,22]. In the hypothalamus, insulin and leptin act synergistically, and their reciprocal control is essential for feeding as well as for the complex energy homeostasis of the whole body [9,22].

Importantly, aberrations of both insulin and leptin signal transduction are implicated in serious metabolic disorders such as type 2 diabetes (T2DM) and obesity. Insulin resistance is a characteristic feature shared by both T2DM and obesity; in this condition, the impaired cellular response to insulin in peripheral tissues can also cause central insulin resistance through the production of toxic lipids that are able to cross the blood–brain barrier and promote neurodegeneration [23]. In addition, in obesity, inflammatory reactions implicate reduced insulin sensitivity of hypothalamic neurons and favor the development of insulin resistance at both central and peripheral levels [9,20]. The condition of leptin resistance, observed in both obese animals and humans, can also attenuate cellular insulin sensitivity, and, in turn, insulin resistance can impair leptin signaling, thus leading to the frequent co-existence of T2DM and obesity [21,22].

It was demonstrated that elevated activity or overexpression of PTP1B can underlie the development of these complex metabolic conditions through the attenuation of both insulin and leptin signals [11,13,24,25]. Indeed, PTP1B-knockout mice exhibited a phenotype ascribable to both enhanced leptin and insulin signaling, characterized by improved sensitivity of target tissues to insulin and resistance to diet-induced obesity, without any alterations in growth or viability [26,27]. Conversely, in obese animals, attenuated or defective hypothalamic leptin signaling proved to be linked to increased food intake and reduced energy expenditure [28]. Interestingly, it was demonstrated that neuronal PTP1B is critically involved in the regulation of body weight by controlling both insulin and leptin signaling in the brain, whereas muscle- or liver-specific deletion of PTP1B resulted in improved glucose homeostasis without protecting animals from weight gain induced by a high-fat diet [11,12,18,19,29]. Moreover, it is worth noting that low-grade inflammation is a feature of both T2DM and obesity, and the expression of PTP1B was shown to be linked to that of pro-inflammatory cytokines such as tumor necrosis factor-α (TNF-α) and interleukins IL-1 and IL-6 [30,31]. Therefore, an indirect implication of PTP1B in obesity could also occur through the modulation of the cellular inflammatory response [10].

PTP1B upregulation can also contribute to the development of diabetic complications, such as cardiovascular pathologies, retinopathy, nephropathy, and foot ulcers, by promoting endothelial dysfunction, inflammation, oxidative stress, and apoptosis [8,15].

Overall, a large body of compelling evidence indicates that PTP1B deletion or inhibition can be an effective strategy to improve glucose homeostasis, control body weight, and prevent hyperglycemia-induced complications, thus prompting the search for PTP1B inhibitors as novel antidiabetic and anti-obesity agents [3,10,25,32,33,34].

Moreover, recent studies on how insulin functions in the brain revealed that this hormone also plays strategic roles in neuronal survival, synaptic plasticity, and memory/learning processes. A direct implication of insulin resistance in the pathogenesis of severe neurodegenerative diseases, such as Alzheimer’s disease (AD), was demonstrated [9,23,35,36]. Insulin can exert a wide action spectrum in the brain, from feeding behavior to olfaction and cognitive processes, and, in fact, IR is expressed not only in the hypothalamus but also in other brain regions, such as the hippocampus, olfactory bulb, and cortex [9]. Leptin receptors are also expressed in the hippocampus, and leptin signaling has been shown to be involved in memory and cognitive processes. In fact, alterations in brain leptin signaling were observed in AD, especially in hippocampal neurons, demonstrating that leptin resistance is another crucial pathogenic factor in the development of this disease [37,38].

Strong evidence implicates PTP1B in AD pathogenesis through multiple mechanisms; once again, the main roles of the enzyme are related to the down-regulation of neuronal insulin and leptin signals, which can result in impaired cognitive functions [36]. In addition, it was demonstrated that, besides IR, IRS, and JAK2, another substrate of neuronal PTP1B is the brain-derived neurotrophic factor (BDNF) receptor TrkB, which is implicated in AD etiology [39]. In fact, BDNF has neuroprotective function and is a major regulator of synaptic plasticity; reduced BDNF levels were found in AD brains, which could result from PTP1B upregulation [36,39].

Moreover, the production and accumulation of amyloid-β peptide (Aβ) oligomers in the brain, which are key events responsible for the synaptic and cognitive alterations typical of AD, can induce the activation of TNF-*α*.

*α* receptors, which, in turn, promote neuronal endoplasmic reticulum (ER) stress, an important neurodegenerative mechanism capable of causing detrimental effects on synapse stability and cognition [36,40]. Neuronal ER stress was shown to induce PTP1B upregulation, exacerbating PTP1B-mediated insulin resistance and leptin resistance in both central and peripheral tissues [36]. In addition, in microglia, the upregulation of this phosphatase proved to be positively linked to chronic neuroinflammation, which is another important pathogenetic factor in AD [36,41]. Therefore, PTP1B deregulation can play a central role in linking inflammation, ER stress, insulin resistance, and leptin resistance, thus representing a probable causal connection between obesity, T2DM, and neurodegeneration. These findings strongly suggest that PTP1B inhibition could provide a novel disease-modifying strategy for the treatment of AD.

Another potential application of PTP1B inhibitors as therapeutic agents could arise from the oncogenic role of the enzyme. Unbalanced protein tyrosine phosphorylation can underlie the development of many cancer types. In addition, the well-established oncogenic role of numerous PTKs, recently several different PTPs were also recognized to promote tumorigenesis by being able to facilitate and support the proliferation, survival, migration, and invasiveness of different neoplastic phenotypes, as well as tumoral angiogenesis [42]. In particular, numerous studies were performed on PTP1B, which was shown to act as an oncogenic protein in specific cell types [4,42,43,44]. PTP1B overexpression was detected in breast cancer at several development stages, representing both an early pathogenic event and a condition that supports and promotes the growth and spread of this kind of tumor [42,44,45]. In particular, PTP1B was shown to play a selective role as a positive modulator of oncogenic signaling induced by the receptor tyrosine kinase HER2/ErbB-2 [44,46,47]. An oncogenic synergy between PTP1B and HER2/ErbB-2 was observed in both breast and ovarian carcinomas [45,47]. In mice with ErbB-2-induced breast cancer, PTP1B deficiency or inhibition delayed tumor progression, protected against lung metastases, and triggered earlier apoptosis by down-regulating both Ras-MAPK and PI3K-Akt signaling pathways [46].

Moreover, a correlation between PTP1B overexpression and cancer growth was found in other tumor types, such as gastric and prostate carcinomas [48,49]. The increased expression as well as the elevated intrinsic activity of PTP1B were found in human colorectal cancer (CRC) tissues, suggesting a promoting role for this phosphatase in the formation and progression of this kind of cancer. PTP1B overexpression was shown to be correlated with worse patient survival, whereas its downregulation resulted in reduced proliferation, adhesion, migration, and invasiveness of CRC cells [50].

However, the role of PTP1B in cancer has been debated. The ability of this enzyme to negatively regulate several receptor-type PTKs related to oncogenesis, including the epidermal growth factor receptor (EGFR) and IR, suggested that it could act as a tumor suppressor. In line with this hypothesis, PTP1B deletion proved to accelerate lymphomagenesis in p53-null mice [51]. On the other hand, PTP1B-knockout animals did not show an increased incidence of tumor development [45]. Therefore, it is plausible that PTP1B can act as a tumor promoter or suppressor depending on the tissue type and the coexistence of aberrations in other factors involved in tumorigenesis [45].

## 3. PTP1B as a Molecular Target for Designing Novel Drugs

On the basis of the advancing knowledge about the pathophysiological roles of PTP1B, in the last two decades a large variety of inhibitors of this phosphatase have been designed and evaluated in preclinical studies, validating the inhibition of this enzyme as an effective approach to counteract specific pathogenetic mechanisms underlying the etiology of several human diseases [3,4,5,33,36,52]. However, only a few PTP1B inhibitors have entered clinical trials so far. In fact, the development of small-molecule inhibitors of PTP1B as drug candidates proved to be a challenging task, mainly due to certain structural features of these enzymes, such as the polar nature and high degree of homology of their catalytic sites. These features complicate the identification of active site-directed inhibitors endowed with appropriate selectivity and cell membrane permeability [6,36], so much so that some doubts were initially raised about the druggability of PTPs [53,54,55]. Nevertheless, in an effort to find drug-like PTP inhibitors, the design of inhibitors targeting non-catalytic regions with specific structural characteristics and/or bearing lipophilic moieties was effectively explored [3,56,57,58,59].

The full-length PTP1B protein consists of 435 amino acids, with the catalytic domain located in the N-terminal, at the bottom of a deep crevice, and a disordered loop at the C-terminal domain. In the active site, the phosphate recognition loop (P-loop, His214-Ser222) defines a cavity able to accommodate phosphotyrosine (pTyr) residues of the substrates and includes the “PTP signature motif” (I/V)HCXAGXGR(S/T). This latter sequence contains some conserved amino acids shared by other cysteine-based PTPs, such as Cys215 and Arg221, which are essential for the dephosphorylation mechanism catalyzed by the enzyme. The catalytic cleft is surrounded by loops that are involved in substrate recognition and catalytic dephosphorylation, such as the WPD loop, Q loop, and YRD loop. Following the substrate binding to the P-loop, the flexible WPD loop (Thr177-Pro185) undergoes a conformational change by moving toward the pTyr residue of the substrate and folding over it, thus allowing the catalytic residue Asp181 to assume an optimal orientation and act as an acid in the first step of the catalytic mechanism [59,60,61]. The residue Gln262 of the Q loop also participates in the catalytic mechanism, and, in addition, the YRD loop is involved in substrate recognition, especially through interactions established by Tyr46 and Asp48 with the substrate [60,62].

Various aryl phosphates with different structures can be accommodated in the flexible PTP1B active site; however, a single pTyr residue does not ensure high affinity for the enzyme, and, thus, other pTyr residues of the substrate contribute to binding the enzyme more efficiently through interactions with subsites close to the catalytic cavity. In particular, a secondary non-catalytic pTyr-binding site, which is lined by Tyr20, Arg24, Ala27, Phe52, Arg254, Met258, and Gly259, participates in substrate recognition and, being not conserved in most PTPs, is considered an attractive binding site for the identification of selective PTP1B inhibitors [60,63]. In fact, a number of bidentate ligands able to bind both the catalytic and secondary non-catalytic sites of the target were designed as PTP1B inhibitors endowed with interesting properties [2,62,64,65,66].

Moreover, significant conformational changes in the PTP1B active site can be induced by the reversible oxidation of the catalytic residue Cys215 to a sulphenyl-amide state by reactive oxygen species, leading to the reversible inactivation of the enzyme. Interestingly, small molecule inhibitors capable of stabilizing the inactive oxidized form of PTP1B and improving insulin and leptin signaling were recently identified [67].

The positively charged catalytic site of PTP1B requires that competitive inhibitors possess acidic structural moieties able to act as pTyr-mimetics, such as aryl phosphonates, carboxylates, or their isosteres; this may lead to highly ionizable compounds that could possess sub-optimal pharmacokinetics, thus explaining the in vivo unsatisfactory outcomes obtained with several potent inhibitors directed to the PTP1B active site.

A promising alternative arises from the presence of allosteric regions on the surface of PTP1B; effective interactions with these sites can be established by less polar ligands, potentially endowed with more favorable pharmacokinetics than conventional catalytic site-directed inhibitors. Moreover, these non-catalytic regions comprise non-conserved amino acid residues, which provide an opportunity for developing selective PTP1B inhibitors [55,68,69,70,71,72].

**Figure 1:**
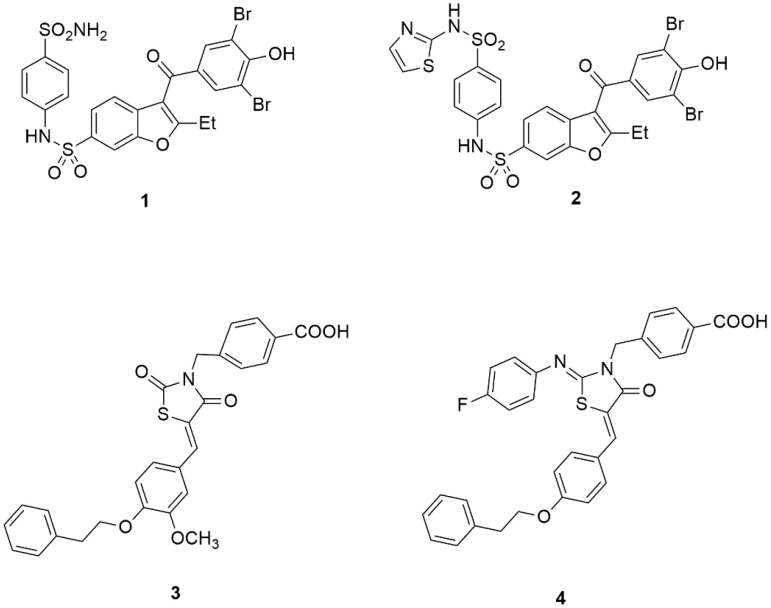
Representative examples of non-competitive PTP1B inhibitors Refs. [68,71,72].

A crystallographic study on non-competitive benzbromarone inhibitors of PTP1B (such as compounds **1**, **2**, Figure 1) led to the identification of an allosteric site located ∼20 Å from the catalytic site of the target enzyme, between helices *α*3 (Glu186-Glu200) and *α*6 (Ala264-Ile281), characterized by a hydrophobic cavity lined by non-conserved residues such as Leu192, Phe196, and Phe280 [68]. The binding of inhibitors to this site was shown to prevent the closure of the catalytic WPD loop by impeding the interactions between helix α7 (Ser285–Ser295) and helices α3 and α6, which are important to stabilize the active closed form of PTP1B, and consequently blocking the enzyme in an inactive conformation [68,73,74].

Another binding site for non-competitive inhibitors was found to be positioned between the *β*-sheet, including Leu71 and Lys73, and a lipophilic pocket delimited by a loop consisting of amino acids Leu 204–Pro210; this latter cavity is connected to the PTP1B catalytic site (Cys215) via a *β*-strand consisting of only five amino acids. Appropriately functionalized 4-thiazolidinone derivatives (such as compounds **3**, **4**, Figure 1), able to establish effective interactions with this allosteric site, proved to act as non-competitive or mixed-type PTP1B inhibitors [71,72]. Interestingly, the opportunity to bind this novel non-catalytic region was also shown to be a promising tool for the design of multiple ligands selectively directed to both PTP1B and aldose reductase, this latter being another enzyme implicated in the development of diabetes and its complications [75,76,77].

On the whole, these findings strongly suggest that, among the feasible approaches to face the difficulties correlated with the development of PTP1B inhibitors, allostery could provide medicinal chemists with a successful strategy to design new compounds endowed with adequate selectivity and cell permeability.

A particularly representative example of an allosteric PTP1B inhibitor is trodusquemine (MSI-1436), a natural aminosterol that proved to be a safe, bioavailable compound and even entered clinical trials. The advancing knowledge of its biological properties allows the therapeutic potential of non-competitive PTP1B inhibitors to be validated and can be assumed as a starting point for developing new drug candidates.

## 4. Identification of Trodusquemine and Its Mechanism of PTP1B Inhibition

**Figure 2:**
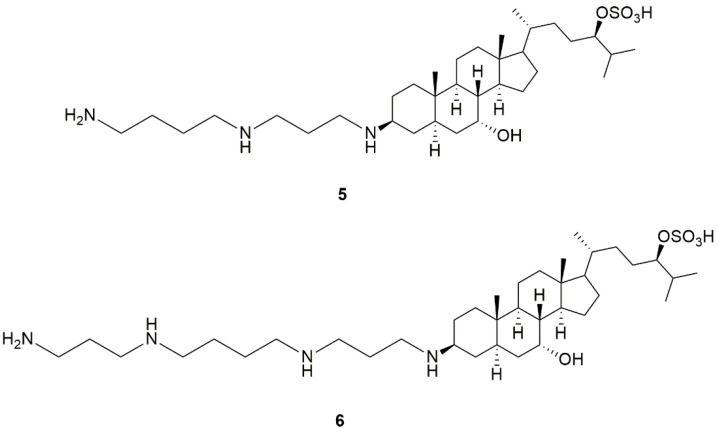
Structures of squalamine (**5**) and trodusquemine (**6**, MSI-1436).

The identification of squalamine (**5**, Figure 2), the first aminosterol isolated from the dogfish shark Squalus acanthias, which exhibited in vitro and in vivo broad-spectrum antimicrobial activity along with antiangiogenic and antitumor properties [78,79], attracted interest and prompted the search for both naturally occurring and synthetic analogues [80,81,82,83]. Among them, trodusquemine (**6**, MSI-1436, Figure 2), which was found in dogfish shark liver, emerged as an antimicrobial agent endowed with greater effectiveness than squalamine [80]. Trodusqumine is a spermine metabolite of cholesterol that shares typical structural features with other characterized aminosterols, such as a steroid skeleton with trans AB/BC/CD ring junctions, a polyamine chain in position 3*β*, a hydroxyl group in 7*α*, and a sulphated 2-methyleptanyl chain in position 17. It differs from squalamine for the spermine sidechain in 3, which possesses an additional propylamino distal portion and an increased positive charge compared with the spermidine chain of squalamine.

Krishnan et al. provided an important piece of knowledge by demonstrating that trodusquemine acts as a reversible, non-competitive PTP1B inhibitor capable of binding a C-terminal non-catalytic region of the enzyme [84]. The full-length PTP1B_435_ is the main cellular form, but shorter variants are also expressed and often used for biochemical studies [68,84]. Experimental data reported by Krishnan et al. revealed that trodusquemine can bind a long PTP1B_405_ form with seven-fold higher affinity compared to a shorter PTP1B_321_ variant in which the regulatory non-catalytic C-terminal segment is absent [84]. The binding of trodusquemine to PTP1B_405_ showed an inhibitor/enzyme stoichiometric ratio of 2 and a positive cooperativity between two PTP1B binding sites. Moreover, the binding of this inhibitor induced a conformational change in PTP1B, leading to a more compact structure of the enzyme in which the flexible, disordered C-terminus moved toward the N-terminus. NMR spectroscopy showed that residues included in the helix *α*9 of the C-terminal segment, such as Arg371, Arg373, and Ser393, were perturbed upon trodusquemine interaction, constituting the primary site involved in the inhibitor binding. The second binding site involved in this two-site mechanism was identified in a region located in close proximity to helix *α*7 and including residues Leu299, Pro310, and Pro311, adjacent to the catalytic segment, and partially overlapped with the allosteric site that was individuated by Wiesmann et al. [68]. Therefore, it was proposed that the binding of trodusquemine to the primary site in the C-terminal helix *α*9 can induce a conformational change in the PTP1B protein, which brings helix *α*7 close to helices *α*3 and *α*6, thus forming the secondary site available to bind an additional molecule of the inhibitor. The cooperative binding to both of these non-catalytic sites can result in the stabilization of an inactive conformation of PTP1B [84].

To our knowledge, further details regarding the formation of the trodusquemine/PTP1B complex have not been reported so far, and no results matching trodusquemine were found in the Protein Data Bank (https://www.rcsb.org, accessed on 26 April 2023).

## 5. Anti-Obesity and Antidiabetic Actions of Trodusquemine

Serendipitously, in vivo investigations revealed surprising pharmacological properties of trodusquemine, which made it a promising drug candidate for various human pathologies.

In particular, several studies carried out in different animal models provided a consistent body of evidence that definitely demonstrated the significant anti-obesity and antidiabetic activities of this PTP1B inhibitor, supporting its potential as an agent for the treatment of metabolic disorders.

Zasloff et al. first described the effects of trodusquemine in wild-type and genetically obese rodents [85]. The parenteral administration of the aminosterol in rats and mice resulted in prolonged body weight reduction by suppressing food and fluid intake while at the same time keeping the treated animals healthy. Moreover, in ob/ob mice, the chronic treatment with trodusquemine over a four-month period safely controlled blood glucose and cholesterol levels. After peripheral injection in mice, trodusquemine was distributed to several tissues, including the brain, demonstrating the capability of this aminosterol to cross the blood–brain barrier. Furthermore, when administered intracerebrally, via direct introduction into the third ventricle of rodents, the aminosterol provided comparable effects at doses remarkably lower than those required systemically, thus suggesting that its anorectic properties could be related to direct central actions [85].

A related study, performed in rodents, confirmed that trodusquemine acts in the brain, particularly in the paraventricular hypothalamic nucleus, and demonstrated that it causes body weight loss not only by reducing food intake but also by increasing energy expenditure [86]. In fact, trodusquemine was able to control specific hypothalamic neuronal pathways that mediate both feeding behavior and energy balance by reducing the expression of the hypothalamic orexigenic neuropeptides AGRP and NPY [86]. Interestingly, the treatment with trodusquemine did not activate compensatory mechanisms physiologically associated with fasting, such as hyperphagia or reduced energy expenditure [85].

Structure–activity relationship studies, performed through the evaluation of synthetic analogues in mice, were consistent with the concept that the observed anorectic effect of trodusquemine is attributable to a specific mechanism of action. In fact, the inversion of configuration at C-3, C-7, or C-20, as well as modifications of the spermine chain in 3 or the sulphated moiety in 17, generally resulted in a drastic reduction in the activity [85]. Interestingly, squalamine was found not to affect food intake or body weight [85], thus highlighting the crucial influence of the polyamine chain on the pharmacological activity of these aminosterols.

Takahashi et al. studied the effects of trodusquemine in genetically leptin-deficient Lep^ob/ob^ mice, a well-known mammalian model of severe insulin resistance, obesity, and hepatic steatosis [87]. The intraperitoneal administration of trodusquemine in these animals resulted in a reduction in both body weight and fat mass, once again by suppressing appetite and promoting energy expenditure. In addition, it restored liver size and histology, unlike food restriction, which alone could not produce appreciable effects on hepatic steatosis. These findings were related to the capability of trodusquemine to suppress lipogenic genes and stimulate lipolytic genes involved in hepatic lipid metabolism. Moreover, trodusquemine was able to act as an insulin sensitizer by improving the hepatic response to the hormone and normalizing blood glucose, insulin, and lipid levels [87].

A pivotal preclinical study was carried out in a murine diet-induced obesity model, which provided fundamental findings to better define the potential of trodusquemine as an anti-obesity agent [88]. In this satisfactory model of human obesity, the administration of trodusquemine suppressed appetite and caused sustained body weight loss in a fat-specific manner. Indeed, it brought about a reduction in total body fat content as well as decreased adipocyte size and lipid content in both white and brown adipose tissues without a loss of lean body mass. According to earlier findings, trodusquemine-induced body weight loss did not activate compensatory mechanisms, such as reduced energy expenditure, therefore resulting in a prolonged anti-obesity effect. Moreover, the treatment with the aminosterol resulted in reduced plasma levels of both insulin and leptin [88]. Importantly, this study defined for the first time the molecular mechanism of the observed anti-obesity and insulin-sensitizing effects of trodusquemine, which were shown to result from the potent and selective inhibition of PTP1B. In fact, MSI-1436 was shown to act as a non-competitive inhibitor of this phosphatase, with an IC_50_ value of 1 μM and more than 200-fold selectivity over the highly homologous enzyme TC-PTP [88]. As a result of this inhibitory activity, trodusquemine significantly augmented the insulin-stimulated phosphorylation of the IR subunit *β*, in both HepG2 cell cultures and rat hypothalamic tissue, thus proving to act as an insulin-sensitizing agent. Moreover, a noteworthy increase in the phosphorylation of hypothalamic STAT-3 was observed, indicating that trodusquemine also improved leptin signaling [88]. In addition, it was demonstrated that this aminosterol can suppress food intake without altering dopamine reuptake in vivo, although it showed inhibitory capability toward the transporter responsible for dopamine reuptake in the course of an in vitro screening [88,89].

Taken together, these findings demonstrated that PTP1B inhibition is the crucial mechanism responsible for the pharmacological effects elicited by trodusquemine by enhancing both insulin and leptin signaling in central and peripheral tissues [88].

The essential role played by PTP1B inhibition in the anti-obesity and antidiabetic activities of trodusquemine was confirmed by studies on the LIM domain only 4 (LMO4) protein, an endogenous negative regulator of PTP1B. It was found that the neuron-specific ablation of LMO4 in mice results in uncontrolled activity of PTP1B, thus causing hyperglycemia, impaired leptin signaling in the hypothalamus, and obesity; conversely, the intracerebroventricular administration of trodusquemine in LMO4-deficient mice restored central leptin signaling and improved the control exerted by this hormone on blood insulin levels [90,91].

The observed capability of trodusquemine to enhance whole-body insulin sensitivity and counteract mechanisms linked to hepatic steatosis attracted interest in further investigating the potential of this compound for the treatment of metabolic disorders, including non-alcoholic fatty liver disease (NAFLD), which is frequently coexistent with T2DM and obesity. In particular, a close correlation between NAFLD and T2DM was demonstrated since these metabolic diseases share some pathogenetic mechanisms, including insulin resistance. Moreover, NAFLD increases the risk of developing T2DM, and, conversely, a higher prevalence of NAFLD occurs in T2DM patients [92]. Accumulation and storage of free fatty acids (FFAs) in non-adipose tissues, such as the liver, cause lipotoxicity, which can lead to cellular dysfunction and apoptotic cell death by impairing insulin signaling and promoting ER stress, oxidative stress, and mitochondrial dysfunction. It was found that the FFA-induced overexpression of PTP1B is involved in the pathophysiological mechanisms governing these cellular events [93]. Indeed, liver-specific deletion of this phosphatase was shown to protect against FFA-induced ER stress and improve insulin sensitivity [94]. Accordingly, trodusquemine was shown to prevent palmitate/oleate-induced lipotoxicity/lipoapoptosis in HepG2 cells by reducing intracellular lipid accumulation, attenuating ER/oxidative stress, and improving mitochondrial dynamics [93]. Similarly, trodusquemine proved to protect tunicamycin-treated HepG2 cells from ER stress and associated mitochondrial dysfunction [95]. ER stress and FFA-induced lipotoxicity in liver tissue represent major hallmarks of both T2DM and NAFLD; moreover, ER stress was recognized as a fundamental pathogenic factor implicated in inflammation as well as in central and peripheral resistance to both insulin and leptin [96,97,98]. Therefore, these findings further support the validity of PTP1B inhibition to control key cellular mechanisms underlying pathological conditions shared by T2DM, obesity, and other metabolic disorders.

Bourebaba et al. also reported studies performed in adipose stem progenitor cells (ASCs) isolated from horses affected by equine metabolic syndrome (EMS) with the objective of investigating the effects of trodusquemine, and therefore of PTP1B inhibition, on several mechanisms underlying the involvement of adipose tissue in insulin resistance. Trodusquemine was found to improve adipogenic differentiation in ASCs, reduce both ER and oxidative stress by decreasing ROS and NO production, normalize FFA intracellular concentrations, and modulate mitochondrial dynamics. Overall, these findings highlighted that PTP1B inhibition can counteract adipocyte dysfunction, which plays a crucial role in the development of insulin resistance in obesity and the metabolic syndrome [99,100].

The meaningful results obtained from the above-described ex vivo/in vivo studies prompted the clinical evaluation of trodusquemine as an anti-obesity and antidiabetic agent. The following Phase I trials can be found at https://clinicaltrials.gov, 26 April 2023: NCT00509132, NCT00806338, and NCT00606112, which were carried out in diabetic and/or obese patients; however, results from completed trials are not yet available. The preliminary presentation of results reported that the intravenous administration of trodusquemine was well tolerated in humans without causing serious adverse effects; this candidate drug showed good pharmacokinetics and significantly improved glycemic control in overweight/obese T2DM patients [55,101,102]. Unfortunately, Phase II trials have not been started yet, apparently due to a lack of financial resources [55,101].

## 6. Anticancer Activity of Trodusquemine

In order to investigate the correlation between PTP1B inhibition and other activities of trodusquemine, Krishnan et al. evaluated the effects of this aminosterol in MCF10A mammary epithelial cells expressing a chimeric HER2/ErbB2 form. Trodusquemine inhibited the proliferation and migration of these cells, although it was ineffective on HER2-negative tumor cells [84]. These results proved that this aminosterol can act by attenuating the PTP1B-induced HER2 signaling amplification in cancer cells [84], in agreement with previous studies that demonstrated a correlation between PTP1B overexpression and HER2/Erb2-positive breast cancer growth and invasiveness [46,47].

Moreover, in a murine model of HER-2 positive breast cancer, trodusquemine proved to control tumorigenesis by reducing tumor growth and blocking the development of metastasis to the lung. Importantly, this study showed, both ex vivo and in vivo, that the ability of trodusquemine to control HER2-induced tumorigenesis and malignancy is specifically related to PTP1B inhibition, once again demonstrating that this enzyme is the key molecular target of trodusquemine [84].

A phase I clinical trial was initiated to evaluate trodusquemine in metastatic breast cancer (NCT02524951); however, to our knowledge, it has been terminated and no data are available.

## 7. Trodusquemine-Induced Regenerative Effects in Multiple Tissues

Another promising activity of trodusquemine emerged in a search for small molecules capable of activating tissue regenerative processes [103]. This aminosterol appreciably stimulated the regeneration of adult zebrafish caudal fins after amputation, resulting in the regrowth of morphologically normal fins without signs of malformation in any tissue. In addition, the aminosterol was able to accelerate heart regeneration in adult zebrafish with ventricular damage [103]. The administration of trodusquemine also elicited a similar effect in adult mice, which have poor heart regenerative capacity; in fact, following ischemic injury, the aminosterol reduced infarct scar size and stimulated cardiomyocyte proliferation. Moreover, in these animals, trodusquemine was also able to activate the proliferation of skeletal muscle satellite cells, which are stem cells necessary for tissue regrowth, resulting in complete muscle regeneration without abnormalities [103].

Interestingly, PTP1B deletion in adult zebrafish resulted in effects on heart regeneration similar to those elicited by trodusquemine, suggesting that the inhibition of this phosphatase is the central mechanism underlying the regenerative properties of the aminosterol [103]. In addition, squalamine was found to be completely inactive [103], once again indicating a clear structure/activity relationship regarding the different polyamine chains, which can crucially influence the PTP1B inhibitory capability of these aminosterols and the resulting activities.

The capability of trodusquemine to stimulate regenerative processes in different tissues and species can be considered a noteworthy feature because it suggests that this aminosterol could also be an agent for the treatment of multiple degenerative human diseases.

Impaired wound healing is common in diabetic patients and represents a serious complication that can lead to diabetic foot ulcers and limb amputation [104]. In these complex conditions, damaged tissues are characterized by active inflammation, elevated oxidative stress, high levels of pro-inflammatory cytokines, increased degradation of the extracellular matrix, endothelial dysfunction, microbial infection, and dysregulation of the immune response. Although knowledge of the mechanisms that underlie compromised wound healing in DM is incomplete, compelling evidence indicates that PTP1B overexpression is implicated, especially in endothelial dysfunction, and, consequently, the deletion or inhibition of this phosphatase could ameliorate wound healing and prevent the onset of related diabetic complications [104]. PTP1B negatively controls the activation of endothelial NO synthase (eNOS), which requires protein tyrosine phosphorylation, whereas PTP1B inhibitors can restore eNOS activity, thus exerting an endothelial protective effect [105].

Moreover, it was demonstrated that PTP1B is a negative key regulator of the vascular endothelial growth factor receptor 2 (VEGFR2) [106]. In diabetic conditions, PTP1B is overexpressed through the hyperglycemia-induced activation of nuclear factor-κB (NF-κB) and can reduce endothelial cell proliferation and angiogenesis through the dephosphorylation of VEGFR2, consequently hampering wound healing [107]. PTP1B−/− mice showed improved diabetic wound healing, and, accordingly, in ob/ob mice as well as in streptozotocin-treated wild-type mice, PTP1B inhibition ameliorated symptoms and accelerated the recovery of skin wounds [107]. In addition, it should be taken into account that PTP1B can also dephosphorylate other protein tyrosine kinases that are involved in cell growth, differentiation, and survival, such as the epidermal growth factor receptor, platelet-derived growth factor receptor, IR, and hepatocyte growth factor receptor; therefore, PTP1B inhibitors could exert multiple effects on regenerative processes by controlling different signaling pathways [104].

Another important factor implicated in the impairment of wound healing is ER stress, which is associated with endothelial dysfunction and tissue inflammation [108]. It was demonstrated that PTP1B, which is localized on the cytoplasmatic face of the ER, can act as a crucial regulator of ER stress in various tissues, including endothelium; in fact, the inhibition or deletion of this phosphatase resulted in decreased ER stress [104]. Accordingly, trodusquemine was shown to reduce tunicamycin-induced ER stress both in vivo in mice and ex vivo in mouse arteries and to prevent endothelial dysfunction through the regulation of several kinases [109]. In addition to endothelium-protective effects, trodusquemine also exhibits a broad spectrum of antimicrobial activity, ranging from several bacterial strains to fungi [80], which could contribute to its effectiveness as a therapeutic agent for the prevention or treatment of diabetic foot ulcers. In fact, counteracting the infection of the wound by pathogens can prevent prolonged inflammation and improve wound healing [104].

## 8. The Potential of Trodusquemine for the Treatment of Cardiovascular Diseases

The protective effects on endothelium and regenerative actions of trodusquemine encouraged the evaluation of this aminosterol in cardiovascular diseases (CVDs) in several preclinical studies [110]. Importantly, CVDs are major, serious complications associated with DM and leading causes of mortality in diabetic patients. In fact, metabolic alterations derived from hyperglycemia, obesity, and insulin resistance are strictly linked to increased cellular oxidative stress and chronic low-grade tissue inflammation [111], which lead to endothelial dysfunction and accelerated atherosclerosis, thus aggravating the risk of CVD development.

In a pivotal study, Thompson et al. showed that, in an LDLR−/− mouse model of atherosclerosis under high-fat diet feeding, trodusquemine administration not only reduced body weight and improved glucose homeostasis but also significantly decreased serum cholesterol and triglyceride levels and counteracted atherosclerotic plaque formation [112]. Surprisingly, no significant increase in IR phosphorylation in the aortic tissues of trodusquemine-treated mice was observed, despite the increased phosphorylation of both AMPK*α*1 and Akt, thus suggesting that the observed anti-atherosclerotic actions could be the result of mechanisms independent from the activation of IR. The increased AMPK*α* phosphorylation and the activation of downstream PI3K/Akt/mTORC1 signaling, which are central events of anti-inflammatory cytokine (IL-10) pathways, could significantly contribute to the capability of trodusquemine to counteract low-grade inflammation and prevent atherosclerosis [112]. The same authors previously reported that myeloid-PTP1B knockout mice showed significantly improved glucose homeostasis, decreased serum lipid levels, and were protected against diet-induced atherosclerotic plaque formation. In these animals, similar to trodusquemine-treated mice, enhanced phosphorylation of aortic AMPK*α* and Akt was observed without an appreciable increase in IR phosphorylation [113].

These findings revealed novel features and therapeutic potential of trodusquemine as an agent for the treatment of different chronic inflammatory pathologies, including not only T2DM and obesity but also CVDs, suggesting that multiple mechanisms, some of which are not linked to IR activation, could be elicited by PTP1B inhibition.

In addition, the above-reported studies on the regeneration of myocardial tissues promoted by trodusquemine in both zebrafish and mice [103] revealed effects of this PTP1B inhibitor that could be significant for the treatment of patients following myocardial infarction. Indeed, PTP1B activity was found to be abnormally elevated in the presence of chronic heart failure (HF), and inhibition or deletion of this enzyme improved cardiac and endothelial dysfunction, with beneficial effects on several parameters related to cardiac remodeling and function [114]. Moreover, Nguyen et al. identified cardiac insulin resistance as a condition that occurs before the onset of mitochondrial and systolic dysfunction in a model of pressure overload-induced HF. Cardiac insulin resistance is strictly related to abnormally high activation of PTP1B in the heart, which was associated with systolic dysfunction in both rats and humans, therefore indicating that the enzyme could be a target for inhibitors capable of improving insulin sensitivity and cardiac function in HF [115].

## 9. The Potential of Trodusquemine for the Treatment of Neurodegenerative Disorders

The ER is the fundamental site in which the synthesis, folding, and maturation of most proteins occur. Therefore, ER dysfunction and the resulting ER stress condition are strictly correlated with increased intracellular accumulation of a variety of misfolded or unfolded proteins [99]. A complex unfolded protein response (UPR) is activated by cells to counteract ER stress and restore protein homeostasis. However, if UPR is insufficient or, conversely, chronically activated, it can lead to pathogenic outcomes [97].

A major risk derived from the accumulation of misfolded or unfolded proteins in cells is their tendency to aggregate and cause a variety of deleterious effects. In particular, the formation of insoluble protein aggregates is a typical feature of many neurodegenerative diseases [97].

The aggregation of *α*-synuclein (*α*S), an intrinsically disordered protein that is expressed in neurons, is a distinctive pathogenetic feature of several neurodegenerative disorders, including Parkinson’s disease (PD). A recent study provided meaningful findings regarding the capability of trodusquemine to inhibit multiple steps of the *α*S aggregation process in both neuronal cell cultures and in a well-established Caenorhabditis elegans model of PD [116]. In particular, trodusquemine was shown to inhibit in vitro *α*S aggregation in a dose-dependent manner by impeding both the lipid-induced initiation and the subsequent fibril-catalyzed secondary nucleation steps. These actions proved to be correlated with the capability of the aminosterol to displace *α*S monomers from the surface of both lipid vesicles and fibrils [116]. Moreover, in human SH-SY5Y neuroblastoma cell cultures, trodusquemine prevented the binding of *α*S oligomers to the cell membrane and significantly reduced the cellular toxicity of these oligomers, also through the control of ROS production [116]. Analogously, in C. elegans nematodes genetically engineered to overexpress *α*S in their large muscle cells, the administration of trodusquemine, before or after the onset of the PD phenotype, brought about a significant decrease in the formation of *α*S inclusions in muscle cells by inhibiting protein aggregation and, consequently, reduced the *α*S aggregate-induced toxicity, thus increasing the health and longevity of worms [116].

Interestingly, the same research group reported that squalamine was also able to counteract *α*S aggregation both in vitro and in vivo and to reduce the toxicity of *α*S oligomers by displacing them from cell membranes and blocking the lipid-induced primary nucleation [117,118,119]. On the basis of preclinical studies, recently squalamine entered clinical trials for the treatment of PD as the phosphate salt ENT-01 directed to *α*S aggregates, which accumulate within the enteric nervous system in the early stages of PD and might underlie the gradual accumulation of the protein in other regions of the nervous system, including the brain; the oral administration of ENT-01 resulted in improved bowel function in PD patients, with minimal systemic absorption and no significant adverse effects [102,120].

However, trodusquemine showed greater effectiveness than squalamine in reducing *α*S aggregation, likely due to its more complex mechanism of action, which involves the inhibition of several steps of the aggregation process [102,116,119,121]. Furthermore, it is worth noting that other actions mediated by trodusquemine, such as the activation of insulin and leptin signaling and its ability to stimulate tissue regeneration, could be involved in the increased lifespan observed in both wild-type and PD worms that were treated with this aminosterol [102].

The capability of trodusquemine to inhibit the aggregation of a protein strictly linked to neurodegeneration, along with its established ability to cross the blood–brain barrier and to promote tissue regeneration, highlighted a novel attractive feature of this molecule and prompted similar investigations on different proteins implicated in neurodegenerative diseases.

Aberrant protein aggregation is also a hallmark of AD. In particular, AD is characterized by the formation of amyloid plaques in the brain, which originate from the aggregation of the intrinsically disordered peptide Aβ, especially of its 42-residue form (A*β*42). The formation of Aβ aggregates is a crucial event in the complex and multifactorial pathogenesis of AD and causes multiple neuronal and synaptic dysfunctions, neuroinflammation, and neuronal cell death [122,123]. Soluble Aβ dimers extracted from AD brains were recognized as the smallest synaptotoxic species, which can be sequestered into the amyloid plaque core and are capable of potently impairing synapse function and memory [124].

Trodusquemine was shown to accelerate the aggregation of A*β*42 both in vitro and in an A*β*42-overexpressing C. elegans model of AD, in particular enhancing the rate of Aβ42 aggregation in the secondary nucleation step with minimal effects on the primary nucleation. However, the A*β*42-induced toxicity was significantly reduced in both neuroblastoma SH-SY5Y cells and worms [125]. It was suggested that this effect could result from the considerable reduction in the number of oligomers bound to the cell membranes that was observed in the presence of trodusquemine [125], in agreement with the finding that the susceptibility of neuronal cells to the toxic effects induced by different misfolded peptides is correlated with the oligomer binding to cellular membranes [126]. In addition, trodusquemine proved to interact with A*β*42 oligomers by promoting the formation of aggregates with increased size [125]. Overall, the reduced binding of A*β*42 oligomers to cell membranes, along with the enhanced conversion of these low molecular weight peptides to ordered and less toxic amyloid fibrils, could be responsible for the decrease in neurotoxicity observed in the presence of trodusquemine [125].

The capability of this aminosterol to displace protein oligomers from cell membranes was also shown with other misfolded oligomers, such as the 40-residue form of Aβ (A*β*40), αS, and oligomeric species derived from the bacterial HypF protein. In any case, trodusquemine suppressed the toxicity induced by these different oligomers in neuroblastoma cells through the displacement of the oligomers from cell membranes, which was shown to be a conserved mechanism useful to prevent their aggregation and cytotoxicity, whereas only marginal effects on the structures of the same oligomers were found [121]. It was suggested that counteracting the binding of misfolded peptides to membranes could not only interfere with their aggregation kinetics but also prevent the uptake of these peptides into neuronal cells, consequently abrogating their cytotoxicity [121].

Further studies carried out with trodusquemine and analogous aminosterols confirmed that the displacement of toxic peptide oligomers from cellular membranes is a central mechanism responsible for the suppression of oligomer toxicity associated with neurodegeneration [119,127,128,129,130].

Errico et al. demonstrated that trodusquemine can strongly bind to both large unilamellar vesicles and cultured neuroblastoma cell membranes and induce changes in the bilayer physicochemical properties able to protect membranes from the interaction of toxic misfolded oligomers [127]. Molecular dynamics simulations indicated that trodusquemine is partially inserted into the membrane bilayer, with the steroidal scaffold buried at the interface between the hydrophilic and hydrophobic layers, whereas the positively charged spermine chain was positioned on the hydrophilic surface of the membrane and the sulphate group pointed towards the solvent [127]. In agreement with these findings, the binding affinity of toxic oligomers for membranes was shown to be significantly reduced in the presence of trodusquemine [128].

A recent study, carried out in lipid monolayers used as models of the outer layer model of the plasma membrane, indicated that a trodusquemine-induced reorganization of the membrane lipids could be correlated with the protective effects of the aminosterol against the binding of misfolded peptide oligomers [130]. Moreover, the bilayer composition can influence the binding affinity of both trodusquemine and toxic oligomers, since cholesterol and ganglioside GM1 appeared to exert opposite effects on these interactions, and, in particular, GM1 favored the amyloid toxicity playing a role in neurodegeneration, whereas the efficient binding of trodusquemine to the membrane bilayer was shown to depend on the presence of cholesterol [127,130]. In cultured neuroblastoma cells, trodusquemine was internalized in the cytoplasm and localized mainly in lysosomes. It also showed high affinity for murine myelinated nerve fibers [129]. On this basis, it was suggested that the localization of the aminosterol in lysosomes and the lipid-rich myeline sheath could be critical to controlling pathogenic events that occur in these specific cellular regions in the course of AD and PD, such as neuronal lysosome dysfunction and the accumulation of amyloid neuritic plaques [129].

Similar to trodusquemine, analogous aminosterols, including squalamine, were shown to enhance Aβ42 aggregation by accelerating secondary nucleation, whereas the first step of *α*S aggregation was inhibited by displacing monomers of the protein from the vesicle surfaces, in both cases reducing the availability of toxic monomers/oligomers in neuronal cells. Squalamine was found to be from three-fold to ten-fold less effective than trodusquemine, suggesting that slight differences in the positively charged chain in position 3 of the sterol core can be critical to controlling the binding of peptide oligomers to cellular membranes and therefore reducing oligomer cytotoxicity. Analogously, des-squalamine, lacking the sulphate group in the 17-chain, and *α*-squalamine, which is the 3-epimer of squalamine, were shown to be less active than trodusquemine [119].

Overall, these findings revealed the important potential of trodusquemine to prevent the oligomer-induced cytotoxicity implicated in neurodegeneration and, moreover, suggested that modulating the interactions of misfolded peptide oligomers with cell membranes through small molecule agents could emerge as a novel therapeutic strategy with a significant impact in the treatment of neurodegenerative diseases.

This concept is also supported by the ability of trodusquemine to inhibit PTP1B in the brain. In fact, as reported above, metabolic disorders are frequently present in AD patients, and it is well-established that insulin resistance and T2DM, in which PTP1B activation is crucially implicated, can significantly increase the risk of developing AD. The direct implication of insulin resistance in the synaptic dysfunctions and memory impairment observed in AD led to the description of this latter disease as “type 3 diabetes”, highlighting the critical role played by metabolic alterations in its pathogenesis [23,35,131]. Moreover, inflammation and ER stress, which are commonly observed in AD, can induce PTP1B overexpression via nuclear factor kB (NF-kB) activation in multiple peripheral and central insulin-target tissues [31], thus worsening the insulin resistance condition. PTP1B activation was found to contribute to cognitive impairment by preventing the phosphorylation and consequent inactivation of glycogen synthase kinase 3*β* (GSK3*β*), an enzyme implicated in the formation of cerebral deposits and neuronal death in AD [132]. Recently, Ricke et al. demonstrated that, similar to neuronal PTP1B ablation, the inhibition of PTP1B by trodusquemine improved the response to insulin in the brain, increased GSK3*β* phosphorylation, prevented hippocampal neuronal loss, and ameliorated cognitive performance in hAPP-J20 mice, a model of familial AD [133]. In addition, treatment with this PTP1B inhibitor, but not the neuronal deletion of the enzyme, reduced inflammation in the hippocampus of hAPP-J20 mice [133]. It is worth noting that trodusquemine did not alter brain integrity or cognitive processes in wild-type mice [134].

Trodusquemine was also shown to control other different actions of PTP1B in the brain [135]. Knocking out the endogenous PTP1B inhibitor LMO4 in mice resulted in markedly increased anxiety and impaired fear extinction due to reduced glutamate-mediated endocannabinoid signaling [134,136]. The activation of metabotropic glutamate receptors (mGluR) depends on the phosphorylation of specific tyrosine residues in these receptors. In the absence of the inhibitory control of LMO4, PTP1B was shown to impair glutamatergic signaling by dephosphorylating mGluR5; on the contrary, the treatment of LMO4-knockout mice with the PTP1B inhibitor trodusquemine restored mGluR5 function and relieved the anxiety phenotype of these animals through a restoration of the endocannabinoid signaling in the amygdala [134]. In addition, PTP1B activity was shown to be elevated in wild-type mice repeatedly stressed with corticosteroids; once again, trodusquemine proved to alleviate this stress-induced anxiety phenotype without exerting any effect in non-anxious animals [134]. This study highlighted that PTP1B also plays a role in stress-induced anxiety and, moreover, suggested that the selective anxiolytic action of trodusquemine could be taken into consideration as an additional therapeutic potential of this small molecule PTP1B inhibitor [134,136].

LMO4-deficient mice also displayed schizophrenia-like behaviors, which were shown to be linked to the impairment of endocannabinoid signaling induced by highly activated PTP1B. The treatment with trodusquemine restored the phosphorylation of the BDNF receptor TrkB and, consequently, ameliorated endocannabinoid signaling, thus improving schizophrenia-related deficits [137]. Trodusquemine also proved to counteract schizophrenia-like behaviors induced by ketamine in mice [138]. These studies suggested that trodusquemine might also be considered as a new lead antipsychotic agent potentially useful for the treatment of schizophrenia-like symptoms [138].

However, further studies are still required to validate the efficacy of targeting PTP1B in neurological disorders; in particular, clinical trials would be desirable to assess whether the above-described activities of trodusquemine can also be found in humans.

## 10. Synthetic Analogues of Trodusquemine

Several multistep synthetic methods were developed in order to obtain analogues of trodusquemine and squalamine endowed with pharmacological activities [82,83,139,140].

**Figure 3:**
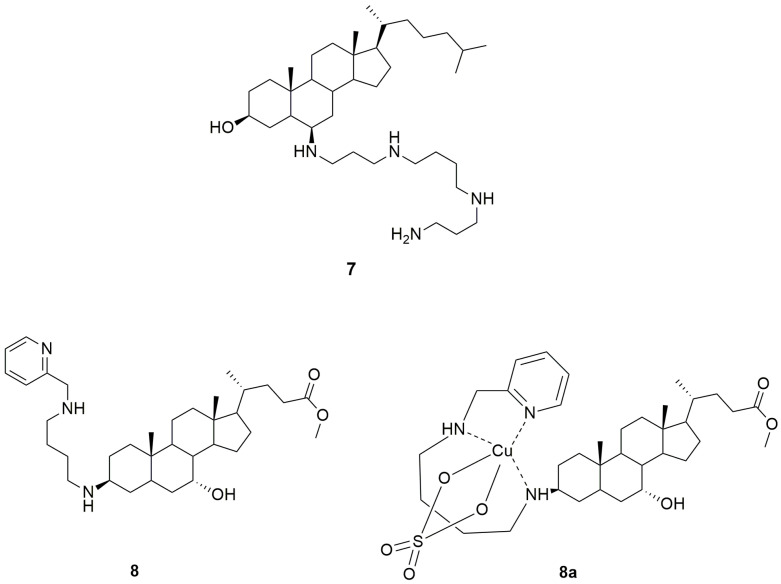
Structures of claramine (**7**), DPM-1001 (**8**), and DPM-1001 copper chelate (**8a**).

Qin et al. reported the synthesis and evaluation of an analogue of trodusquemine, named claramine (**7**, Figure 3), which differs from the lead aminosterol for the 17*β*-alkyl moiety, in which the sulphate group was removed, and for the displacement of both the spermine chain from C-3*β* to C-6*β* and the hydroxyl group from C-7*α* to C-3*β* of the steroid skeleton [139]. Similar to trodusquemine, claramine proved to act as a selective PTP1B inhibitor in both lysed and cultured neuronal cells. Although its PTP1B inhibitory potency was higher in vitro (50% PTP1B inhibition at 0.5 μM concentration) than in intact living cells, claramine elicited insulin-mimetic effects in neuronal cells by enhancing the phosphorylation of IR*β*, Akt, and GSK3*β* and was able to control glycemic levels in a murine diabetes model, together with a stronger but less prolonged appetite suppressing effect than trodusquemine [139]. These findings indicated that claramine can cross the blood–brain barrier, likely exerting its anorectic effect in the hypothalamus, in analogy with trodusquemine. A recent study confirmed the ability of claramine to improve leptin and insulin signaling in the brain [141]. In fact, intracerebroventricular administration of a combination of the glucocorticoid antagonist RU486, which can reduce TC-PTP expression, and claramine synergistically promoted weight loss in obese mice by reducing food intake and fat mass and improving glucose metabolism. Moreover, this pharmacological combination increased energy expenditure by promoting white adipose tissue browning [141]. Analogously, the intranasal administration of the claramine/RU486 combination in obese mice, resulting in direct delivery to the brain, enhanced both leptin and insulin signals, thus inducing weight loss and improving glucose tolerance [141]. It was worth noting that the effects of these two agents are correlated with the specific targeting of both hypothalamic PTP1B and TC-PTP, since they had no additional effect when administered after PTP1B and TC-PTP deletion [141].

Interestingly, claramine was also shown to mitigate the toxicity of different pore-forming agents, such as α-hemolysin and melittin, which can disrupt cell membrane integrity by embedding within the lipid bilayer and causing pore formation [142]. It was suggested that claramine could interact with cell membranes similar to trodusquemine by inserting close to the interface between the hydrophilic and hydrophobic layers of the membrane, whereas the positively charged spermine chain could be positioned on the surface of the bilayer. Consequently, cell membranes become less negatively charged, and a redistribution of membrane lipids occurs, resulting in the modulation of the physical state of the lipid bilayer and protective effects from membrane disrupting proteins [142].

Recently, Krishnan et al. reported another synthetic analogue of trodusquemine, named DPM-1001 (**8**, Figure 3), which was shown to be a potent non-competitive inhibitor of PTP1B (IC_50_ = 100 nM), six-fold more effective than trodusquemine, with marked selectivity for the long PTP1B_405_ form, containing the extended C-terminal segment, over other PTPs. Moreover, this new aminosterol inhibited a trodusquemine-resistant mutant of PTP1B (L192A/S372P), with an IC_50_ value of 1 μM [143]. Compound **8** differs from trodusquemine for the polyamine chain in position 3, which contains a pyridyl head, and for the substituent in position 17 of the steroidal skeleton, in which the sulphate group was replaced by an ester moiety (Figure 3).

The structure of the polyamine chain allows DPM-1001 to specifically coordinate copper ions, forming a stable chelate (**8a**, Figure 3). On the basis of electrospray ionization mass spectrometry (ESI-MS) analysis, it was proposed that DPM-1001 could act as a tridentate ligand, forming two chelate rings, one seven-membered and one five-membered, with a sulphate (or nitrate) anion also involved in the metal complexation [143]. The N^1^-(pyridin-2-ylmethyl)butane-1,4-diamine chain proved to be essential for metal chelation, whereas the steroidal skeleton conferred selectivity towards copper. In fact, DPM-1001 was not able to form complexes with other metals, whereas the non-steroidal analogue N^1^,N^4^-bis(pyridin-2-ylmethyl)butane-1,4-diamine, in which the steroid moiety was replaced by a second pyridine ring, was shown to complex copper as well as several other metals [144]. Moreover, the removal of the N^1^-(pyridin-2-ylmethyl)butane-1,4-diamine chain of compound **8** was sufficient to abolish the ability to chelate copper. Similarly, the replacement of the pyridyl moiety with piperidine or benzene rings, as well as the displacement of the pyridyl nitrogen atom from position 2 to position 3 of the heterocycle, also prevented copper chelation [144].

Interestingly, copper chelate **8a** proved to be able to selectively bind and inhibit PTP1B with higher potency than the uncoordinated ligand **8**, thus providing a novel copper-dependent feature of the inhibition mechanism toward the target enzyme [143].

Krishnan et al. also reported interesting antidiabetic properties of compound **8**. In fact, when administered to high-fat diet-fed mice, DPM-1001 proved to improve both insulin and leptin signaling, bringing about body weight loss and improved glycemic control. Both oral and intraperitoneal administration of DPM-1001 produced the same effects in the treated obese animals [143], highlighting that this aminosterol has improved pharmacokinetics and oral bioavailability compared to the parent trodusquemine, likely due to its less ionizable structure. On these bases, DPM-1001 could be assumed to be a new drug candidate for further evaluation of its antidiabetic and anti-obesity effectiveness [59].

In addition, the copper-chelating ability of DPM-1001 was shown to be useful to attenuate toxic effects associated with the accumulation of copper in fibroblasts derived from patients affected by Wilson’s disease. The oral or intraperitoneal administration of this aminosterol in a murine model of Wilson’s disease reduced copper levels in the liver and brain and ameliorated the symptoms associated with the disease [144]. Therefore, it was suggested that DPM-1001 could also be investigated for the treatment of other pathologic conditions associated with elevated copper levels, including cancer and neurodegenerative diseases [144].

## 11. Conclusions

**Figure 4:**
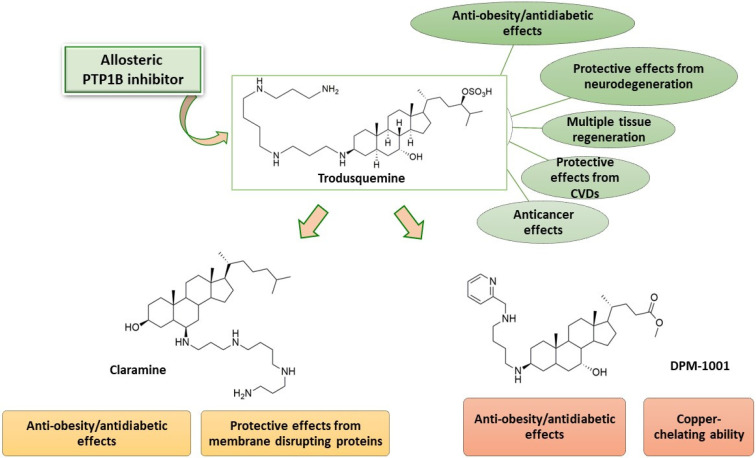
Summary of the biological activities shown by trodusquemine, claramine, and DPM-1001 as PTP1B inhibitors.

Trodusquemine proved to be a promising PTP1B inhibitor with a multifaceted activity profile that has been incompletely studied in clinical trials. A large body of evidence proved that it is not only an efficacious antidiabetic and anti-obesity agent endowed with good pharmacokinetics and safety of use in both animal models and humans, but also to possess great potential for the therapeutic treatment of various other pathologies, such as diabetic complications, cancer, cardiovascular, and neurodegenerative diseases (Figure 4). In particular, taking into account that at present there is an urgent need for efficacious disease-modifying drugs capable of blocking or slowing down the progression of neurodegenerative diseases, it would be worthwhile to study these aspects more thoroughly and also assess their clinical impact. A deeper investigation of recently reported synthetic analogues of trodusquemine, such as claramine and DPM-1001, which showed promising properties in preclinical studies (Figure 4), would also be desirable in order to obtain further significant findings about their therapeutic potential.

Importantly, it was demonstrated that most effects elicited by trodusquemine derive from the inhibition of PTP1B, which was definitely shown to be the central target of this aminosterol. These findings provided further knowledge about this intriguing enzyme by showing its implications in different pathogenetic mechanisms linked to the etiology of several human diseases and, therefore, confirming that it can be an attractive molecular target for the development of new therapeutic agents. Moreover, the results so far acquired corroborate the validity of allosteric inhibition as a key strategy to discover PTP1B inhibitors endowed with drug-like properties, especially in terms of selectivity, bioavailability, and safety. In fact, the difficulties that have hindered the progress of the search for drugs directed at this enzyme so far mainly arise from certain structural features of the PTP1B active site. The surprising biological activities of trodusquemine, as well as investigations carried out on other non-competitive PTP1B inhibitors, emphasized the druggability of PTP1B through its binding to non-catalytic pockets, strongly suggesting that targeting allosteric regions of this enzyme can be a viable tool to overcome the issues related to the development of inhibitors directed to its catalytic domain. Therefore, allostery could be a guiding concept for medicinal chemists in their efforts to design small-molecule PTP1B inhibitors as novel potential drugs.

## Data Availability

Not applicable.

## References

[1] Bennasroune A., Gardin A., Aunis D., Crémel G., Hubert P. (2004). Tyrosine Kinase Receptors as Attractive Targets of Cancer Therapy. Crit. Rev. Oncol. Hemat..

[2] Bialy L., Waldmann H. (2005). Inhibitors of Protein Tyrosine Phosphatases: Next-Generation Drugs?. Angew. Chem. Int..

[3] Zhang S., Zhang Z.Y. (2007). PTP1B as a Drug Target: Recent Developments in PTP1B Inhibitor Discovery. Drug Discov. Today.

[4] Blaskovich M.A.T. (2009). Drug Discovery and Protein Tyrosine Phosphatases. Curr. Med. Chem..

[5] Vintonyak V.V., Waldmann H., Rauh D. (2011). Using Small Molecules to Target Protein Phosphatases. Bioorg. Med. Chem..

[6] Maccari R., Ottanà R. (2012). Low Molecular Weight Phosphotyrosine Protein Phosphatases as Emerging Targets for the Design of Novel Therapeutic Agents. J. Med. Chem..

[7] Alonso A., Sasin J., Bottini N., Friedberg I., Friedberg I., Osterman A., Godzik A., Hunter T., Dixon J., Mustelin T. (2004). Protein Tyrosine Phosphatases in the Human Genome. Cell.

[8] Abdelsalam S.S., Korashy H.M., Zeidan A., Agouni A. (2019). The Role of Protein Tyrosine Phosphatase (PTP)-1B in Cardiovascular Disease and its Interplay with Insulin Resistance. Biomolecules.

[9] Beddows C.A., Dodd G.T. (2021). Insulin on the Brain: The Role of Central Insulin Signalling in Energy and Glucose Homeostasis. J. Neuroendocrinol..

[10] Villamar-Cruz O., Loza-Mejia M.A., Arias-Romero L.E., Camacho-Arroyo I. (2021). Recent Advances in PTP1B Signaling in Metabolism and Cancer. Bioscience Rep..

[11] Zabolotny J.M., Bence-Hanulec K.K., Stricker-Krongrad A., Haj F., Wang Y., Minokoshi Y., Kim Y.B., Elmquist J.K., Tartaglia L.A., Kahn B.B. (2002). PTP1B Regulates Leptin Signal Transduction In Vivo. Dev. Cell.

[12] Cheng A., Uetani N., Simoncic P.D., Chaubey V.P., Lee-Loy A., McGlade C.J., Kennedy B.P., Tremblay M.L. (2002). Attenuation of Leptin Action and Regulation of Obesity by Protein Tyrosine Phosphatase 1B. Dev. Cell.

[13] Zhang Z.Y., Dodd G.T., Tiganis T. (2015). Protein Tyrosine Phosphatases in Hypothalamic Insulin and Leptin Signaling. Trends Pharmacol. Sci..

[14] Bakke J., Haj F.G. (2015). Protein-Tyrosine Phosphatase 1B Substrates and Metabolic Regulation. Semin. Cell Dev. Biol..

[15] Teimouri M., Hosseini H., ArabSadeghabadi Z., Babaei-Khorzoughi R., Gorgani-Firuzjaee S., Meshkani R. (2022). The Role of Protein Tyrosine Phosphatase 1B (PTP1B) in the Pathogenesis of Type 2 Diabetes Mellitus and its Complications. J. Physiol. Biochem..

[16] Saltiel A.R., Kahn C.R. (2001). Insulin Signalling and the Regulation of Glucose and Lipid Metabolism. Nature.

[17] Salmeen A., Andersen J.N., Myers M.P., Tonks N.K., Barford D. (2000). Molecular Basis for the Dephosphorylation of the Activation Segment of the Insulin Receptor by Protein Tyrosine Phosphatase 1B. Mol. Cell..

[18] Delibegovic M., Bence K., Mody N., Hong E.-G., Ko H.J., Kim J.K., Kahn B.B., Neel B.G. (2007). Improved Glucose Homeostasis in Mice with Muscle-Specific Deletion of Protein-Tyrosine Phosphatase 1B. Mol. Cell. Biol..

[19] Delibegovic M., Zimmer D., Kauffman C., Rak K., Hong E., Cho Y., Kim J.K., Kahn B.B., Neel B.G., Bence K.K. (2009). Liver-Specific Deletion of Protein-Tyrosine Phosphatase 1B (PTP1B) Improves Metabolic Syndrome and Attenuates Diet-Induced Endoplasmic Reticulum Stress. Diabetes.

[20] Dodd G.T., Tiganis T. (2017). Insulin Action in the Brain: Roles in Energy and Glucose Homeostasis. J. Neuroendocrinol..

[21] Sainz N., Barrenetxe J., Moreno-Aliaga M.J., Martinez J.A. (2015). Leptin Resistance and Diet-Induced Obesity: Central and Peripheral Actions of Leptin. Metabolism.

[22] Thon M., Hosoi T., Ozawa K. (2016). Possible Integrative Actions of Leptin and Insulin Signaling in the Hypothalamus Targeting Energy Homeostasis. Front. Endocrinol..

[23] de la Monte S.M. (2009). Insulin Resistance and Alzheimer’s Disease. BMB Rep..

[24] Di Paola R., Frittitta L., Miscio G., Bozzali M., Baratta R., Centra M., Spampinato D., Santagati M.G., Ercolino T., Cisternino C. (2002). A Variation in 3’ UTR of hPTP1B Increases Specific Gene Expression and Associates with Insulin Resistance. Am. J. Hum. Genet..

[25] Miranda S., Gonzalez-Rodriguez A., Revuelta-Cervantes J., Rondinone C.M., Valverde A.M. (2010). Beneficial Effects of PTP1B Deficiency on Brown Adipocyte Differentiation and Protection Against Apoptosis Induced by Pro- and Anti-Inflammatory Stimuli. Cell. Sign..

[26] Elchebly M., Payette P., Michaliszyn E., Cromlish W., Collins S., Loy A.L., Normandin D., Cheng A., Himms-Hagen J., Chan C.-C. (1999). Increased Insulin Sensitivity and Obesity Resistance in Mice Lacking the Protein Tyrosine Phosphatase-1B Gene. Science.

[27] Klaman L.D., Boss O., Peroni O.D., Kim J.K., Martino J.L., Zabolotny J.M., Moghal N., Lubkin M., Kim Y.B., Sharpe A.H. (2000). Increased Energy Expenditure, Decreased Adiposity, and Tissue-Specific Insulin Sensitivity in Protein-Tyrosine Phosphatase 1B-Deficient Mice. Mol. Cell. Biol..

[28] Morton G.J., Cummings D.E., Baskin D.G., Barsh G.S., Schwartz M.W. (2006). Central Nervous System Control of Food Intake and Body Weight. Nature.

[29] Bence K.K., Delibegovic M., Xue B., Gorgun C.Z., Hotamisligil G.S., Neel B.G., Kahn B.B. (2006). Neuronal PTP1B Regulates Body Weight, Adiposity and Leptin Action. Nat. Med..

[30] Nieto-Vazquez I., Fernandez-Veledo S., De Alvaro C., Rondinone C.M., Valverde A.M., Lorenzo M. (2007). Protein-Tyrosine Phosphatase 1B-Deficient Myocytes Show Increased Insulin Sensitivity and Protection against Tumor Necrosis Factor-α-Induced Insulin Resistance. Diabetes.

[31] Zabolotny J.M., Kim Y.B., Welsh L.A., Kershaw E.E., Neel B.G., Kahn B.B. (2008). Protein-Tyrosine Phosphatase 1B Expression Is Induced by Inflammation In Vivo. J. Biol. Chem..

[32] Gum R.J., Gaede L.L., Koterski S.L., Heindel M., Clampit J.E., Zinker B.A., Trevillyan J.M., Ulrich R.G., Jirousek M.R., Rondinone C.M. (2003). Reduction of Protein Tyrosine Phosphatase 1B Increases Insulin-Dependent Signalling in ob/ob Mice. Diabetes.

[33] Digenio A., Pham N.C., Watts L.M., Morgan E.S., Jung S.W., Baker B.F., Geary R.S., Bhanot S. (2018). Antisense inhibition of protein tyrosine phosphatase 1B with IONIS-PTP-1BRx improves insulin sensitivity and reduces weight in overweight patients with type 2 diabetes. Diabetes Care.

[34] Singh S., Grewal A.S., Grover R., Sharma N., Chopra B., Dhingra A.K., Arora S., Redhu S., Lather V. (2022). Recent Updates on Development of Protein-Tyrosine Phosphatase 1B Inhibitors for Treatment of Diabetes, Obesity and Related Disorders. Bioorg. Chem..

[35] McNay E.C., Ong C.T., McCrimmon R.J., Cresswell J., Bogan J.S., Sherwin R.S. (2010). Hippocampal Memory Processes Are Modulated by Insulin and High-Fat-Induced Insulin Resistance. Neurobiol. Learn. Mem..

[36] Vieira M.N.N., Lyra e Silva N.M., Ferreira S.T., De Felice F.G. (2017). Protein Tyrosine Phosphatase 1B (PTP1B): A Potential Target for Alzheimer’s Therapy?. Front. Aging Neurosci..

[37] Bonda D.J., Stone J.G., Torres S.L., Siedlak S.L., Perry G., Kryscio R., Jicha G., Casadesus G., Smith M.A., Zhu X. (2014). Dysregulation of Leptin Signaling in Alzheimer Disease: Evidence for Neuronal Leptin Resistance. J. Neurochem..

[38] Maioli S., Lodeiro M., Merino-Serrais P., Falahati F., Khan W., Puerta E., Codita A., Rimondini R., Ramirez M.J., Simmons A. (2015). Alterations in Brain Leptin Signalling in Spite of Unchanged CSF Leptin Levels in Alzheimer’s Disease. Aging Cell..

[39] Ozek C., Kanoski S.E., Zhang Z.Y., Grill H.J., Bence K.K. (2014). Protein-Tyrosine Phosphatase 1B (PTP1B) Is a Novel Regulator of Central Brain-Derived Neurotrophic Factor and Tropomyosin Receptor Kinase B (Trkb) Signaling. J. Biol. Chem..

[40] Jeon Y., Lee S., Kim S., Kwon Y., Kim K., Chung C.G., Lee S., Lee S.B., Kim H. (2017). Neuroprotective Effects of Protein Tyrosine Phosphatase 1B Inhibition against ER Stress-Induced Toxicity. Mol. Cells.

[41] Song G.J., Jung M., Kim J.H., Park H., Rahman M.H., Zhang S., Zhang Z.Y., Park D.H., Kook H., Lee I.K. (2016). A Novel Role for Protein Tyrosine Phosphatase 1B as a Positive Regulator of Neuroinflammation. J. Neuroinflamm..

[42] Lazo J.S., McQueeney K.E., Burnett J.C., Wipf P., Sharlow E.R. (2018). Small Molecule Targeting of PTPs in Cancer. Int. J. Biochem. Cell B.

[43] Sastry S.K., Elferink L.A. (2011). Checks and Balances: Interplay of RTKS and PTPs in Cancer Progression. Biochem. Pharmacol..

[44] Yu M., Liu Z., Liu Y., Zhou X., Sun F., Liu Y., Li L., Hua S., Zhao Y., Gao H. (2019). PTP1B Markedly Promotes Breast Cancer Progression and is Regulated by MiR-193a-3p. FEBS J..

[45] Lessard L., Stuible M., Tremblay M.L. (2010). The Two Faces of PTP1B in Cancer. Biochim. Biophys. Acta Proteins Proteom..

[46] Julien S.G., Dubè N., Read M., Penney J., Paquet M., Han Y., Kennedy B.P., Muller W.J., Tremblay M.L. (2007). Protein Tyrosine Phosphatase 1B Deficiency or Inhibition Delays ErbB2-Induced Mammary Tumorigenesis and Protect from Lung Metastasis. Nat. Gen..

[47] Bentires-Alj M., Neel B.G. (2007). Protein-Tyrosine Phosphatase 1B Is Required for HER2/Neu-Induced Breast Cancer. Cancer Res..

[48] Wang J., Chen X.-, Liu B., Zhu Z. (2010). Suppression of PTP1B in Gastric Cancer Cells in Vitro Induces a Change in the Genome-Wide Expression Profile and Inhibits Gastric Cancer Cell Growth. Cell Biol. Int..

[49] Lessard L., Labbé D.P., Deblois G., Beǵin L.R., Hardy S., Mes-Masson A.M., Saad F., Trotman L.C., Giquère V., Tremblay M.L. (2012). PTP1B Is an Androgen Receptor-Regulated Phosphatase that Promotes the Progression of Prostate Cancer. Cancer Res..

[50] Hoekstra E., Das A.M., Swets M., Cao W., van der Woude C.J., Bruno M.J., Peppelenbosch M.P., Kuppen P.J.K., Ten Hagen T.L., Fuhler G.M. (2016). Increased PTP1B Expression and Phosphatase Activity in Colorectal Cancer Results in a More Invasive Phenotype and Worse Patient Outcome. Oncotarget.

[51] Dubé N., Bourdeau A., Heinonen K.M., Cheng A., Loy A.L., Tremblay M.L. (2005). Genetic Ablation of Protein Tyrosine Phosphatase 1B Accelerates Lymphomagenesis of p53-Null Mice through the Regulation of B-cell Development. Cancer Res..

[52] Tamrakar A.K., Maurya C.K., Ray A.K. (2014). PTP1B Inhibitors for Type 2 Diabetes Treatment: A Patent Review (2011–2014). Expert Opin. Ther. Pat..

[53] Hajduk P.J., Huth J.R., Tse C. (2005). Predicting Protein Druggability. Drug Discov. Today.

[54] Cheng A.C., Coleman R.G., Smyth K.T., Cao Q., Soulard P., Caffrey D.R., Salzberg A.C., Huang E.S. (2007). Structure-Based Maximal Affinity Model Predicts Small-Molecule Druggability. Nat. Biotechnol..

[55] Ghattas M.A., Raslan N., Sadeq A., Sorkhy M.A., Atatreh N. (2016). Druggability Analysis and Classification of Protein Tyrosine Phosphatase Active Sites. Drug Des. Devel. Ther..

[56] Popov D. (2011). Novel Protein Tyrosine Phosphatase 1B Inhibitors: Interaction Requirements for Improved Intracellular Efficacy in Type 2 Diabetes Mellitus and Obesity Control. Biochem. Biophys. Res. Commun..

[57] Stanford S.M., Bottini N. (2017). Targeting Tyrosine Phosphatases: Time to End the Stigma. Trends Pharmacol. Sci..

[58] Köhn M. (2020). Turn and Face the Strange: A New View on Phosphatases. ACS Cent. Sci..

[59] Liu R., Mathieu C., Berthelet J., Zhang W., Dupret J.-M., Rodrigues Lima F. (2022). Human Protein Tyrosine Phosphatase 1B (PTP1B): From Structure to Clinical Inhibitor Perspectives. Int. J. Mol. Sci..

[60] Nichols A.J., Mashal R.D., Balkan B. (2006). Toward the Discovery of Small Molecule PTP1B Inhibitors for the Treatment of Metabolic Diseases. Drug. Dev. Res..

[61] Koren S., Fantus I.G. (2007). Inhibition of the Protein Tyrosine Phosphatase PTP1B: Potential Therapy for Obesity, Insulin Resistance and Type-2 Diabetes Mellitus. Best Pract. Res. Clin. Endocrinol. Metab..

[62] Scapin G., Patel S.B., Becker J.W., Wang Q., Desponts C., Waddleton D., Skorey K., Cromlish W., Bayly C., Therien M. (2003). The Structural Basis for the Selectivity of Benzotriazole Inhibitors of PTP1B. Biochemistry.

[63] Puius Y.A., Zhao Y., Sullivan M., Lawrence D.S., Almo S.C., Zhang Z.Y. (1997). Identification of a Second Aryl Phosphate-Binding Site in Protein-Tyrosine Phosphatase 1B: A Paradigm for Inhibitor Design. Proc. Natl. Acad. Sci. USA.

[64] Combs A.P. (2010). Recent Advances in the Discovery of Competitive Protein Tyrosine Phosphatase 1B Inhibitors for the Treatment of Diabetes, Obesity and Cancer. J. Med. Chem..

[65] Maccari R., Ottanà R., Ciurleo R., Paoli P., Manao G., Camici G., Laggner C., Langer T. (2009). Structure-Based Optimization of Benzoic Acids as Inhibitors of Protein Tyrosine Phosphatase 1B and Low Molecular Weight Protein Tyrosine Phosphatase. ChemMedChem.

[66] Ottanà R., Maccari R., Amuso S., Wolber G., Schuster D., Herdlinger S., Manao G., Camici G., Paoli P. (2012). New 4-[(5-arylidene-2-arylimino-4-oxo-3-thiazolidinyl)methyl]Benzoic Acids Active as Protein Tyrosine Phosphatase Inhibitors Endowed with Insulinomimetic Effect on Mouse C2C12 Skeletal Muscle Cells. Eur. J. Med. Chem..

[67] Krishnan N., Bonham C.A., Rus I.A., Shrestha O.K., Gauss C.M., Haque A., Tocilj A., Joshua-Tor L., Tonks N.K. (2018). Harnessing Insulin- And Leptin-Induced Oxidation of PTP1B for Therapeutic Development. Nat. Commun..

[68] Wiesmann C., Barr K.J., Kung J., Zhu J., Erlanson D.A., Shen W., Fahr B.J., Zhong M., Taylor L., Randal M. (2004). Allosteric Inhibition of Protein Tyrosine Phosphatase 1B. Nat. Struct. Mol. Biol..

[69] Morishita K., Shoji Y., Tanaka S., Fukui M., Ito Y., Kitao T., Ozawa S., Hirono S., Shirahase H. (2017). Novel Non-Carboxylate Benzoylsulfonamide-Based Protein Tyrosine Phosphatase 1B Inhibitors with Non-Competitive Actions. Chem. Pharm. Bull..

[70] Elhassan R.M., Hou X., Fang H. (2022). Recent Advances in the Development of Allosteric Protein Tyrosine Phosphatase Inhibitors for Drug Discovery. Med. Res. Rev..

[71] Ottanà R., Maccari R., Mortier J., Caselli A., Amuso S., Camici G., Rotondo A., Wolber G., Paoli P. (2014). Synthesis, Biological Activity and Structure-Activity Relationships of New Benzoic Acid-Based Protein Tyrosine Phosphatase Inhibitors Endowed with Insulinomimetic Effects in Mouse C2C12 Skeletal Muscle Cells. Eur. J. Med. Chem..

[72] Ottanà R., Paoli P., Naß A., Lori G., Cardile V., Adornato I., Rotondo A., Graziano A.C.E., Wolber G., Maccari R. (2017). Discovery of 4-[(5-arylidene-4-oxothiazolidin-3-yl)methyl]benzoic Acid Derivatives Active as Novel Potent Allosteric Inhibitors of Protein Tyrosine Phosphatase 1B: In Silico Studies and in Vitro Evaluation as Insulinomimetic and Anti-Inflammatory Agents. Eur. J. Med. Chem..

[73] Olmez E.O., Alakent B. (2011). Alpha7 Helix Plays an Important Role in the Conformational Stability of PTP1B. J. Biomol. Struct. Dyn..

[74] Schneider R., Beumer C., Simard J.R., Grütter C., Rauh D. (2013). Selective Detection of Allosteric Phosphatase Inhibitors. J. Am. Chem. Soc..

[75] Maccari R., Del Corso A., Paoli P., Adornato I., Lori G., Balestri F., Cappiello M., Naß A., Wolber G., Ottanà R. (2018). An Investigation on 4-Thiazolidinone Derivatives as Dual Inhibitors of Aldose Reductase and Protein Tyrosine Phosphatase 1B, in the Search for Potential Agents for the Treatment of Type 2 Diabetes Mellitus and Its Complications. Bioorg. Med. Chem. Lett..

[76] Ottanà R., Paoli P., Cappiello M., Nguyen T.N., Adornato I., Del Corso A., Genovese M., Nesi I., Moschini R., Naß A. (2021). In Search for Multi-Target Ligands as Potential Agents for Diabetes Mellitus and its Complications—A Structure-Activity Relationship Study on Inhibitors of Aldose Reductase and Protein Tyrosine Phosphatase 1B. Molecules.

[77] Maccari R., Wolber G., Genovese M., Sardelli G., Talagayev V., Balestri F., Luti S., Santi A., Moschini R., Del Corso A. (2023). Designed Multiple Ligands for the Treatment of Type 2 Diabetes Mellitus and its Complications: Discovery of (5-Arylidene-4-Oxo-2-Thioxothiazolidin-3-Yl)alkanoic Acids Active as Novel Dual-Targeted PTP1B/AKR1B1 Inhibitors. Eur. J. Med. Chem..

[78] Moore K.S., Wehrli S., Rodert H., Rogers M., Forrest J.N., McCrimmon D., Zasloff M. (1993). Squalamine: An Aminosterol Antibiotic from the Shark. Proc. Nat. Acad. Sci. USA.

[79] Sills A.K., Williams J.I., Tyler B.M., Epstein D.S., Sipos E.P., Davis J.D., McLane M.P., Pitchford S., Cheshire K., Gannon F.H. (1998). Squalamine Inhibits Angiogenesis and Solid Tumor Growth in Vivo and Perturbs Embryonic Vasculature. Cancer Res..

[80] Rao M.N., Shinnar A.E., Noecker L.A., Chao T.L., Feibush B., Snyder B., Sharkansky I., Sarkahian A., Zhang X., Jones S.R. (2000). Aminosterols from the Dogfish Shark Squalus Acanthias. J. Nat. Prod..

[81] Shu Y., Jones S.R., Kinney W.A., Selinsky B.S. (2002). The Synthesis of Spermine Analogs of the Shark Aminosterol Squalamine. Steroids.

[82] Salmi C., Loncle C., Vidal N., Laget M., Letourneux Y., Brunel J.M. (2008). Antimicrobial Activities of 3-Amino- and Polyaminosterol Analogues of Squalamine and Trodusquemine. J. Enzym. Inhib. Med. Chem..

[83] Salmi C., Loncle C., Vidal N., Letourneux Y., Brune J.M. (2008). New Stereoselective Titanium Reductive Amination Synthesis of 3-Amino and Polyaminosterol Derivatives Possessing Antimicrobial Activities. Eur. J. Med. Chem..

[84] Krishnan N., Koveal D., Miller D.H., Xue B., Akshinthala S.D., Kragelj J., Ringkjøbing Jensen M., Gauss C.M., Page R., Blackledge M. (2014). Targeting the Disordered C-Terminus of PTP1B with an Allosteric Inhibitor. Nat. Chem. Biol..

[85] Zasloff M., Williams J.I., Chen Q., Anderson M., Maeder T., Holroyd K., Jones S., Kinney W., Cheshire K., McLane M. (2001). A Spermine-Coupled Cholesterol Metabolite from the Shark with Potent Appetite Suppressant and Antidiabetic Properties. Int. J. Obes..

[86] Ahima R.S., Patel H.R., Takahashi N., Qi Y., Hileman S.M., Zasloff M.A. (2002). Appetite Suppression and Weight Reduction by a Centrally Active Aminosterol. Diabetes.

[87] Takahashi N., Qi Y., Patel H.R., Ahima R.S. (2004). A Novel Aminosterol Reverses Diabetes and Fatty Liver Disease in Obese Mice. J. Hepatol..

[88] Lantz K.A., Emeigh Hart S.G., Planey S.L., Roitman M.F., Ruiz-White I.A., Wolfe H.R., McLane M.P. (2010). Inhibition of PTP1B by Trodusquemine (MSI-1436) Causes Fat-specific Weight Loss in Diet-induced Obese Mice. Obesity.

[89] Roitman M.F., Wescott S., Cone J.J., McLane M.P., Wolfe H.R. (2010). MSI-1436 Reduces Acute Food Intake without Affecting Dopamine Transporter Activity. Pharmacol. Biochem. Behav..

[90] Pandey N.R., Zhou X., Qin Z., Zaman T., Gomez-Smith M., Keyhanian K., Anisman H., Brunel J.M., Stewart A.F.R., Chen H.H. (2013). The LIM Domain only 4 Protein Is a Metabolic Responsive Inhibitor of Protein Tyrosine Phosphatase 1B that Controls Hypothalamic Leptin Signaling. J. Neurosci..

[91] Pandey N.R., Zhou X., Zaman T., Cruz S.A., Qin Z., Lu M., Keyhanian K., Brunel J.M., Stewart A.F.R., Chen H.H. (2014). LMO4 Is Required to Maintain Hypothalamic Insulin Signaling. Biochem. Bioph. Res. Commun..

[92] Gastaldelli A., Cusi K. (2019). From NASH to Diabetes and from Diabetes to NASH: Mechanisms and Treatment Options. JHEP Rep..

[93] Bourebaba L., Łyczko J., Alicka M., Bourebaba N., Szumny A., Fal A.M., Marycz K. (2020). Inhibition of Protein-Tyrosine Phosphatase PTP1B and LMPTP Promotes Palmitate/Oleate-Challenged HepG2 Cell Survival by Reducing Lipoapoptosis, Improving Mitochondrial Dynamics and Mitigating Oxidative and Endoplasmic Reticulum Stress. J. Clin. Med..

[94] Agouni A., Mody N., Owen C., Czopek A., Zimmer D., Bentires-Alj M., Bence K.K., Delibegović M. (2011). Liver-specific Deletion of Protein Tyrosine Phosphatase (PTP) 1B Improves Obesity- and Pharmacologically Induced Endoplasmic Reticulum Stress. Biochem. J..

[95] Bourebaba L., Komakula S.S.B., Weiss C., Adrar N., Marycz K. (2023). The PTP1B Selective Inhibitor MSI-1436 Mitigates Tunicamycin-Induced ER Stress in Human Hepatocarcinoma Cell Line through XBP1 Splicing Modulation. PLoS ONE.

[96] Ron D., Walter P. (2007). Signal Integration in the Endoplasmic Reticulum Unfolded Protein Response. Nat. Rev. Mol. Cell Biol..

[97] Chambers J.E., Marciniak S.J. (2014). Cellular Mechanisms of Endoplasmic Reticulum Stress Signaling in Health and Disease. 2. Protein Misfolding and ER Stress. Am. J. Physiol. Cell Physiol..

[98] Ariyasu D., Yoshida H., Hasegawa Y. (2017). Endoplasmic Reticulum (ER) Stress and Endocrine Disorders. Int. J. Mol. Sci..

[99] Bourebaba L., Kornicka-Garbowska K., Al Naem M., Röcken M., Łyczko J., Marycz K. (2021). MSI-1436 Improves EMS Adipose Derived Progenitor Stem Cells in the Course of Adipogenic Differentiation through Modulation of ER Stress, Apoptosis, and Oxidative Stress. Stem. Cell Res. Ther..

[100] Kornicka-Garbowska K., Bourebaba L., Röcken M., Marycz K. (2021). Inhibition of Protein Tyrosine Phosphatase Improves Mitochondrial Bioenergetics and Dynamics, Reduces Oxidative Stress, and Enhances Adipogenic Differentiation Potential in Metabolically Impaired Progenitorstem Cells. Cell Commun. Signal.

[101] Fiedler L.R. (2018). Inhibiting the Inhibitors, PTP1B as a Therapeutic Target in Myocardial Infarction. Heart Res. Open J..

[102] Limbocker R., Errico S., Barbut D., Knowles T.P.J., Vendruscolo M., Chiti F., Zasloff M. (2022). Squalamine and Trodusquemine: Two Natural Products for Neurodegenerative Diseases, from Physical Chemistry to the Clinic. Nat. Prod. Rep..

[103] Smith A.M., Maguire-Nguyen K.K., Rando T.A., Zasloff M.A., Strange K.B., Yin V.P. (2017). The Protein Tyrosine Phosphatase 1B Inhibitor MSI-1436 Stimulates Regeneration of Heart and Multiple Other Tissues. NPJ Regen. Med..

[104] Figueiredo A., Leal E.C., Carvalho E. (2020). Protein Tyrosine Phosphatase 1B Inhibition as a Potential Therapeutic Target for Chronic Wounds in Diabetes. Pharmacol. Res..

[105] Vercauteren M., Remy E., Devaux C., Dautreaux B., Henry J.P., Bauer F., Mulder P., Hooft van Huijsduijnen R., Bombrun A., Thuillez C. (2006). Improvement of Peripheral Endothelial Dysfunction by Protein Tyrosine Phosphatase Inhibitors in Heart Failure. Circulation.

[106] Lanahan A.A., Lech D., Dubrac A., Zhang J., Zhuang Z.W., Eichmann A., Simons M. (2014). PTP1b Is a Physiologic Regulator of Vascular Endothelial Growth Factor Signaling in Endothelial Cells. Circulation.

[107] Zhang J., Li L., Li J., Liu Y., Zhang C.Y., Zhang Y., Zen K. (2015). Protein Tyrosine Phosphatase 1B Impairs Diabetic Wound Healing through Vascular Endothelial Growth Factor Receptor 2 Dephosphorylation. Arterioscler. Thromb. Vasc. Biol..

[108] Schürmann C., Goren I., Linke A., Pfeilschifter J., Frank S. (2014). Deregulated Unfolded Protein Response in Chronic Wounds of Diabetic Ob/Ob Mice: A Potential Connection to Inflammatory and Angiogenic Disorders in Diabetes-Impaired Wound Healing. Biochem. Biophys. Res. Commun..

[109] Thiebaut P.A., Delile E., Coquerel D., Brunel J.M., Renet S., Tamion F., Richard V. (2018). Protein Tyrosine Phosphatase 1B Regulates Endothelial Endoplasmic Reticulum Stress; Role in Endothelial Dysfunction. Vascul. Pharmacol..

[110] Krenz M. (2022). Friend or foe? Unraveling the Complex Roles of Protein Tyrosine Phosphatases in Cardiac Disease and Development. Cell. Signal..

[111] Schmidt M.I., Saad M.J.A., Duncan B.B. (2005). Subclinical Inflammation and Obesity, Diabetes and Related Disorders. Drug Discov. Today Dis. Mech..

[112] Thompson D., Morrice N., Grant L., Le Sommer S., Lees E.K., Mody N., Wilson H.M., Delibegovic M. (2017). Pharmacological Inhibition of Protein Tyrosine Phosphatase 1B Protects Against Atherosclerotic Plaque Formation in the LDLR−/− Mouse Model of Atherosclerosis. Clin. Sci..

[113] Thompson D., Morrice N., Grant L., Le Sommer S., Ziegler K., Whitfield P., Mody N., Wilson H.M., Delibegovic M. (2017). Myeloid Protein Tyrosine Phosphatase 1B (PTP1B) Deficiency Protects Against Atherosclerotic Plaque Formation in the Apoe^-/-^ Mouse Model of Atherosclerosis with Alterations in IL10/AMPKα Pathway. Mol. Metab..

[114] Gomez E., Vercauteren M., Kurtz B., Ouvrard-Pascaud A., Mulder P., Henry J.P., Besnier M., Waget A., Hooft Van Huijsduijnen R., Tremblay M.L. (2012). Reduction of Heart Failure by Pharmacological Inhibition or Gene Deletion of Protein Tyrosine Phosphatase 1B. J. Mol. Cell. Cardiol..

[115] Nguyen T.D., Schwarzer M., Schrepper A., Amorim P.A., Blum D., Hain C., Faerber G., Haendeler J., Altschmied J., Doenst T. (2018). Increased Protein Tyrosine Phosphatase 1B (PTP1B) Activity and Cardiac Insulin Resistance Precede Mitochondrial and Contractile Dysfunction in Pressure-Overloaded Hearts. J Am Heart Assoc..

[116] Perni M., Flagmeier P., Limbocker R., Cascella R., Aprile F.A., Galvagnion C., Heller G.T., Meisl G., Chen S.W., Kumita J.R. (2018). Multistep Inhibition of α-Synuclein Aggregation and Toxicity in Vitro and in Vivo by Trodusquemine. ACS Chem. Biol..

[117] Perni M., Galvagnion C., Maltsev A., Meisl G., Müller M.B.D., Challa P.K., Kirkegaard J.B., Flagmeier P., Cohen S.I.A., Cascella R. (2017). A Natural Product Inhibits the Initiation of a-Synuclein Aggregation and Suppresses Its Toxicity. Proc. Natl. Acad. Sci. USA.

[118] Perni M., van der Goot A., Limbocker R., van Ham T.J., Aprile F.A., Xu C.K., Flagmeier P., Thijssen K., Sormanni P., Fusco G. (2021). Comparative Studies in the A30P and A53T a-Synuclein C. Elegans Strains to Investigate the Molecular Origins of Parkinson’s Disease. Front. Cell Dev. Biol..

[119] Limbocker R., Staats R., Chia S., Ruggeri F.S., Mannini B., Xu C.K., Perni M., Cascella R., Bigi A., Sasser L.R. (2021). Squalamine and its Derivatives Modulate the Aggregation of Amyloid-b and a-Synuclein and Suppress the Toxicity of Their Oligomers. Front. Neurosci..

[120] Hauser R.A., Sutherland D., Madrid J.A., Rol M.A., Frucht S., Isaacson S., Pagan F., Maddux B.N., Li G., Tse W. (2019). Targeting Neurons in the Gastrointestinal Tract to Treat Parkinson’s Disease. Clin. Park. Relat. Disord..

[121] Limbocker R., Mannini B., Ruggeri F.S., Cascella R., Xu C.K., Perni M., Chia S., Chen S.W., Habchi J., Bigi A. (2020). Trodusquemine Displaces Protein Misfolded Oligomers from Cell Membranes and Abrogates Their Cytotoxicity through a Generic Mechanism. Commun. Biol..

[122] Ballard C., Gauthier S., Corbett A., Brayne C., Aarsland D., Jones E. (2011). Alzheimer’s disease. Lancet.

[123] Kreiser R.P., Wright A.K., Block N.R., Hollows J.E., Nguyen L.T., LeForte K., Mannini B., Vendruscolo M., Limbocker R. (2020). Therapeutic Strategies to Reduce the Toxicity of Misfolded Protein Oligomers. Int. J. Mol. Sci..

[124] Shankar G.M., Li S., Mehta T.H., Garcia-Munoz A., Shepardson N.E., Smith I., Brett F.M., Farrell M.A., Rowan M.J., Lemere C.A. (2008). Amyloid-β Protein Dimers Isolated Directly from Alzheimer’s Brains Impair Synaptic Plasticity and Memory. Nat. Med..

[125] Limbocker R., Chia S., Ruggeri F.S., Perni M., Cascella R., Heller G.T., Meisl G., Mannini B., Habchi J., Michaels T.C.T. (2019). Trodusquemine Enhances Aβ42 Aggregation but Suppresses its Toxicity by Displacing Oligomers from Cell Membranes. Nat. Commun..

[126] Evangelisti E., Cascella R., Becatti M., Marrazza G., Dobson C.M., Chiti F., Stefani M., Cecchi C. (2016). Binding affinity of amyloid oligomers to cellular membranes is a generic indicator of cellular dysfunction in protein misfolding diseases. Sci. Rep..

[127] Errico S., Lucchesi G., Odino D., Muscat S., Capitini C., Bugelli C., Canale C., Ferrando R., Grasso G., Barbut D. (2020). Making Biological Membrane Resistant to the Toxicity of Misfolded Protein Oligomers: A Lesson from Trodusquemine. Nanoscale.

[128] Errico S., Ramshini H., Capitini C., Canale C., Spaziano M., Barbut D., Calamai M., Zasloff M., Oropesa-Nuñez R., Vendruscolo M. (2021). Quantitative Measurement of the Affinity of Toxic and Nontoxic Misfolded Protein Oligomers for Lipid Bilayers and of its Modulation by Lipid Composition and Trodusquemine. ACS Chem. Neurosci..

[129] Capitini C., Pesce L., Fani G., Mazzamuto G., Genovese M., Franceschini A., Paoli P., Pieraccini G., Zasloff M., Chiti F. (2022). Studying the Trafficking of Labelled Trodusquemine and its Application as Nerve Marker for Light-Sheet and Expansion Microscopy. FASEB J..

[130] Barletti B., Lucchesi G., Muscat S., Errico S., Barbut D., Danani A., Zasloff M., Grasso G., Chiti F., Caminati G. (2023). Reorganization of the Outer Layer of a Model of the Plasma Membrane Induced by a Neuroprotective Aminosterol. Colloids Surf. B.

[131] Bosco D., Fava A., Plastino M., Montalcini T., Pujia A. (2011). Possible Implications of Insulin Resistance and Glucose Metabolism in Alzheimer’s Disease Pathogenesis. J. Cell. Mol. Med..

[132] Llorens-Martín M., Jurado J., Hernandez F., Avila J. (2014). GSK-3β, a Pivotal Kinase in Alzheimer Disease. Front. Mol. Neurosci..

[133] Ricke K.M., Cruz S.A., Qin Z., Farrokhi K., Sharmin F., Zhang L., Zasloff M.A., Stewart A.F.R., Chen H.H. (2020). Neuronal Protein Tyrosine Phosphatase 1B Hastens Amyloid β-Associated Alzheimer’s Disease in Mice. J. Neurosci..

[134] Olloquequi J., Cano A., Sanchez-Lopez E., Carrasco M., Verdaguer E., Fortuna A., Folch J., Bulló M., Auladell C., Camins A. (2022). Protein Tyrosine Phosphatase 1B (PTP1B) as a Potential Therapeutic Target for Neurological Disorders. Biomed. Pharmacother..

[135] Krishnan N., Tonks N.K. (2015). Anxious Moments for the Protein Tyrosine Phosphatase PTP1B. Trends Neuros..

[136] Qin Z., Zhou X., Pandey N.R., Vecchiarelli H.A., Stewart C.A., Zhang X., Lagace D.C., Brunel J.M., Be´ı¨que J.C., Stewart A.F.R. (2015). Chronic Stress Induces Anxiety via an Amygdalar Intracellular Cascade that Impairs Endocannabinoid Signaling. Neuron.

[137] Qin Z., Zhang L., Cruz S.A., Stewart A.F.R., Chen H.H. (2020). Activation of Tyrosine Phosphatase PTP1B in Pyramidal Neurons Impairs Endocannabinoid Signaling by Tyrosine Receptor Kinase Trkb and Causes Schizophrenia-Like Behaviors in Mice. Neuropsychopharmacology.

[138] Qin Z., Zhang L., Zasloff M.A., Stewart A.F.R., Chen H.H. (2021). Ketamine’s schizophrenia-like effects are prevented by targeting PTP1B. Neurobiol. Dis..

[139] Qin Z., Pandey N.R., Zhou X., Stewart C.A., Hari A., Huang H., Stewart A.F.R., Brunel J.M., Chen H.H. (2015). Functional Properties of Claramine: A Novel PTP1B Inhibitor and Insulin-Mimetic Compound. Biochem. Biophys. Res. Commun..

[140] Blanchet M., Borselli D., Rodallec A., Peiretti F., Vidal N., Bolla J.M., Digiorgio C., Morrison K.R., Wuest W.M., Brunel J.M. (2018). Claramines: A New Class of Broad-Spectrum Antimicrobial Agents with Bimodal Activity. ChemMedChem.

[141] Dodd G.T., Xirouchaki C.E., Eramo M., Mitchell C.A., Andrews Z.B., Henry B.A., Cowley M.A., Tiganis T. (2019). Intranasal Targeting of Hypothalamic PTP1B and TCPTP Reinstates Leptin and Insulin Sensitivity and Promotes Weight Loss in Obesity. Cell Rep..

[142] Kreiser R.P., Wright A.K., Sasser L.R., Rinauro D.J., Gabriel J.M., Hsu C.M., Hurtado J.A., McKenzie T.L., Errico S., Albright J.A. (2022). A Brain-Permeable Aminosterol Regulates Cell Membranes to Mitigate the Toxicity of Diverse Pore-Forming Agents. ACS Chem. Neurosci..

[143] Krishnan N., Konidaris K.F., Gasser G., Tonks N.K. (2018). A Potent, Selective, and Orally Bioavailable Inhibitor of the Protein-Tyrosine Phosphatase PTP1B Improves Insulin and Leptin Signaling in Animal Models. J. Biol. Chem..

[144] Krishnan N., Felice C., Rivera K., Pappin D.J., Tonks N.K. (2018). DPM-1001 Decreased Copper Levels and Ameliorated Deficits in a Mouse Model of Wilson’s Disease. Genes Dev..

